# Whole Genome Sequence Analysis of Weight Loss in 16 972 Participants With COPD Reveals Novel Risk Loci in *DRAIC* and *RFX3*

**DOI:** 10.1002/jcsm.70293

**Published:** 2026-04-23

**Authors:** Joe W. Chiles, Alison Rocco, Vinodh Srinivasasainagendra, Harry B. Rossiter, Richard Casaburi, Anna Thalacker‐Mercer, J. Michael Wells, Emily S. Wan, Edwin K. Silverman, Michael H. Cho, Craig P. Hersh, Bruce M. Psaty, Sina A. Gharib, Yan Gao, George T. O'Connor, Leslie A. Lange, Stephen S. Rich, Ani W. Manichaikul, R. Graham Barr, Victor E. Ortega, Deborah A. Meyers, Albert V. Smith, Hemant K. Tiwari, Merry‐Lynn N. McDonald

**Affiliations:** ^1^ Division of Pulmonary, Allergy, and Critical Care Medicine University of Alabama at Birmingham Birmingham Alabama USA; ^2^ Department of Biostatistics, School of Public Health University of Alabama at Birmingham Birmingham Alabama USA; ^3^ The Lundquist Institute for Biomedical Innovation at Harbor‐UCLA Medical Center Torrance California USA; ^4^ Department of Cell, Developmental and Integrative Biology University of Alabama at Birmingham Birmingham Alabama USA; ^5^ Channing Division of Network Medicine Brigham and Women's Hospital Boston Massachusetts USA; ^6^ VA Boston Healthcare System Boston Massachusetts USA; ^7^ Cardiovascular Health Research Unit, Department of Medicine University of Washington Seattle Washington USA; ^8^ Division of Pulmonary, Critical Care, and Sleep Medicine University of Washington Seattle Washington USA; ^9^ Jackson Heart Study University of Mississippi Medical Center Jackson Mississippi USA; ^10^ Department of Medicine Boston University School of Medicine Boston Massachusetts USA; ^11^ Department of Biomedical Informatics University of Colorado‐Anschutz Aurora Colorado USA; ^12^ Department of Genome Sciences University of Virginia Charlottesville Virginia USA; ^13^ Departments of Medicine and Epidemiology Columbia University Medical Center New York New York USA; ^14^ Division of Pulmonary Medicine, Department of Medicine Mayo Clinic Phoenix Arizona USA; ^15^ Department of Internal Medicine Mayo Clinic Scottsdale Arizona USA; ^16^ Department of Biostatistics University of Michigan Ann Arbor Michigan USA

**Keywords:** cachexia, COPD, genomics, wasting, weight loss

## Abstract

**Background:**

Chronic obstructive pulmonary disease (COPD) is associated with musculoskeletal comorbidities, including cachexia. Weight loss (WL) is the major criterion for cachexia and increases risk for mortality in COPD. Risk factors for WL in COPD are incompletely understood. We performed this whole genome sequencing (WGS) analysis to identify genetic risk variants for WL in COPD.

**Methods:**

We studied 16 972 participants from the Trans‐Omics for Precision Medicine (TOPMed) Initiative and All of Us Research Program. COPD was diagnosed using spirometry in TOPMed, while diagnosis codes were used in All of Us. WL was defined as WL ≥ 5% or a final body mass index (BMI) < 20 kg/m^2^. WGS data came from white blood cells in all cohorts. Single‐variant testing was conducted on both race‐ and study‐stratified cohorts and in a cosmopolitan, ancestry‐independent manner using GENESIS in TOPMed and SAIGE in All of Us. SAIGE‐GENE+ gene‐based analyses were performed on race‐stratified and cosmopolitan cohorts. Single variant meta‐analyses were conducted using METAL within (B/AA and NHW analyses of Black/African–American and non‐Hispanic white participants, respectively) and across racial groups (COSMO). Rare variant gene‐based results were combined using Fisher's method. Transcriptomic effects were predicted using MetaXcan. We used the GWAS Catalogue to analyse for colocalization with other related traits.

**Results:**

Two single variants were associated with WL in COPD among All of Us participants: one intronic variant in *HCN1* in Black/African–American participants (chr5:45271359:TACACAC:T, odds ratio with 95% confidence interval (OR (CI_95_)) = 2.43(1.78–3.31), *p* = 1.95 × 10^−8^) and one intergenic variant between *PPP4R2* and *PDZRN3* in the cosmopolitan and NHW cohorts (chr3:73345901:A:G, OR (CI_95_) = 0.21(0.12–0.35), *p* = 8.84 × 10^−9^ in cosmopolitan and OR (CI_95_) = 0.18(0.10–0.33), *p* = 9.44 × 10^−9^ in NHW). Single‐variant meta‐analysis identified two loci associated with WL in COPD: five variants within *DRAIC* in the B/AA meta‐analysis (lead variant chr15:69571341:A:G, OR (CI_95_) = 1.37(1.23–1.51), *p* = 1.29 × 10^−9^) and two intronic variants within *RFX3* in the cosmopolitan meta‐analysis (lead variant chr9:3390983:T:C, OR (CI_95_) = 1.50(1.31–1.73), *p* = 1.06 × 10^−8^). Rare variants within *RNU6‐565P* (NHW analysis in All of Us; *p* = 2.83 × 10^−7^) and *LOC339298* (B/AA analysis in All of Us, *p* = 1.85 × 10^−6^) were associated with WL in COPD. The *RNU‐565P* signal remained significant after combination with TOPMed results (p_combined_ = 1.23 × 10^−6^). MetaXcan predicted differential expression of *LNC00959* in visceral adipose tissue (*p* = 1.16 × 10^−6^ in COSMO analysis). Colocalization analyses identified genomic associations between BMI and variants in or near *DRAIC*, *RFX3*, *PDZRN3*, and *LINC00959*.

**Conclusions:**

In the first WGS analysis of WL in COPD, we have identified seven novel loci. Further characterization of these loci will validate our findings and improve our understanding of the molecular pathophysiology of this condition.

## 1 Introduction

Chronic obstructive pulmonary disease (COPD) is a complex lung disease caused by exposure to noxious smoke, particles, or gases, as well as by genetic factors [1, 2]. Already one of the top causes of mortality worldwide [3], COPD prevalence is expected to rise throughout the coming decades [4]. Although COPD is diagnosed using lung function testing, muscle wasting and cachexia are extrapulmonary manifestations of this pulmonary disease [5, 6]. For people living with COPD, the presence of cachexia has long been recognized as a risk factor for mortality [5, 6] and is associated with a worse quality of life [7] and functional status [5].

The pathophysiology of cachexia in COPD is incompletely understood. COPD patients with cachexia generally have more severe lung function impairment [5], elevated levels of inflammatory markers [8] and a greater extent of emphysema on radiographic imaging [7]. Weight loss (WL), the major criterion for cachexia, is easily detected and captures most of the association between cachexia and mortality [5, 6]. There is evidence that genetic predisposition influences the development and progression of cachexia. We previously performed a genome‐wide association study (GWAS) of WL in 4308 participants with COPD and found one significant single nucleotide polymorphism (SNP) that did not replicate and gene‐level signals for genetic association with WL in the genes *BAIAP2* and *EFNA2* that did replicate across study populations [9]. The signal for association with variants in *BAIAP2*, which encodes a protein involved in myogenic regulation, was specifically found in non‐Hispanic white (NHW) participants, while the association with variants in *EFNA2*, which encodes a protein‐tyrosine kinase involved in development, was found in African–American participants. Earlier candidate gene studies also identified variants within the *PLA2G2D* gene on chromosome 1, encoding for a phospholipase A2 family member, as being associated with WL [10].

Our previous research examined genotyping array data [9], as opposed to state‐of‐the‐art whole genome sequencing (WGS) data, which provides more comprehensive coverage of the genome. The use of WGS allows for multiple advantages over traditional GWAS studies using SNP arrays and imputation [11], including direct sequencing of variants, less reliance on imputation, which has been occasionally faulty in prior GWAS studies [12], and better detection of rare variants. Rare variants, often identified as those with an alternative allele fraction (AAF) less than 1%, are frequently not in significant linkage disequilibrium (LD) with neighbouring variants [11, 13]. This reduced LD obviates the need to use fine‐mapping to search for causative variant(s) within a locus. Recent studies have emphasized the importance of these rare variants, specifically those in low LD, as they account for significant heritability of complex traits that were not well captured using SNP array data [14].

Through large research longitudinal studies, such as the All of Us Research Program [15] and the studies within the Trans‐Omics for Precision Medicine (TOPMed) Initiative [16], we now have access to a larger number of participants with COPD and WGS data to perform a more robust analysis. In the current report, we present findings from the analysis of WGS data in 16 972 research participants with COPD.

## 2 Methods

### 2.1 WL in Participants With COPD From TOPMed Studies

Participants with COPD from seven TOPMed studies with available whole genome sequence data, pulmonary function testing, smoking history and longitudinal weight measurements were included in analyses. The TOPMed studies included in this analysis were the Cardiovascular Health Study (CHS), Genetic Epidemiology of COPD (COPDGene), Evaluation of COPD to Longitudinally Identify Predictive Surrogate Endpoints (ECLIPSE), the Jackson Heart Study (JHS), the Framingham Heart Study (FHS), the Multi‐Ethnic Study of Atherosclerosis (MESA) Lung Study and SubPopulations and InteRmediate Outcome Measures in COPD Study (SPIROMICS). Full descriptions of TOPMed studies used in this analysis, including references, are available in the Supporting Information section.

#### 2.1.1 COPD

In all TOPMed studies, participants with COPD were over 40 years of age and had a forced expiratory volume in 1 s (FEV_1_) to forced vital capacity (FVC) ratio < 0.7. If postronchodilator spirometry was available (in COPDGene, ECLIPSE, and SPIROMICS), it was used for this determination.

#### 2.1.2 Smoking History

Participants with COPD and at least a 10 pack‐year tobacco exposure were included in analyses with the exception of JHS, where participants with COPD were included if they self‐identified as either a former or current smoker.

#### 2.1.3 WL

In all TOPMed studies, participants were coded as having WL if they experienced a 5% WL between study visits and did not fully regain the lost weight before the last study visit or had a BMI < 20 kg/m^2^ at their final visit. If, at a later visit, a participant was found to have fully regained the weight lost at a previous visit, they were reclassified as a control. Time between visits varied across cohorts: The shortest follow‐up periods occurred in ECLIPSE, where visits were spaced every 6 months, and SPIROMICS, where early follow‐up visits occurred every 1 year but were then spaced further apart, with the other cohorts having generally 5–7 years between visits.

### 2.2 WL in Participants With COPD From the All of Us study

Participants from the All of Us Controlled Tier Dataset version 7 were selected if they had short‐read WGS data available, were aged 40–85 years at the time of their consent and had an International Classification of Diseases 10 (ICD‐10) diagnosis code of J41, J42, J43 or J44. This system for finding cases of COPD in electronic databases has been previously evaluated and shows reasonable sensitivity and specificity [17]. Participants with reported COPD who denied any history of cigarette smoking were excluded from this analysis. All available weight, height and BMI data were extracted from the database for each participant. Body measurements were cleaned for implausibly high or low values (such as a BMI < 10 kg/m^2^ or weight < 20 kg), as well as for outliers by removing values greater than three times the interquartile range either above the 75th percentile or below the 25th percentile for each participant.

Due to differences in weight ascertainment between the electronic medical record data available in All of Us and the discrete nature of TOPMed study visits, WL in All of Us was detected by analysing the last 5 years of available weight and BMI data, starting from the most recent weight record. A 12‐month sliding window was applied, starting with the window spanning from 60 to 48 months before the most recent weight. If this window contained more than two data points, a linear regression line of body weight was fit with time as the independent variable; if the slope value explained a significant amount of the variance in weight and was equal to or greater than 5% body weight per 12 months, a participant was judged to have WL. Similar regression lines were fit starting at each 3‐month window, stopping at 6 months before the last available weight, with the last two windows encompassing only nine and 6 months, respectively. If a participant had at least one window with WL present, their most recently recorded weight was compared with the maximum weight from their WL window; if the maximum weight from the WL window was greater than the final weight, the participant was judged to have evidence of WL; if not, they were judged not to have WL. The last BMI was checked for each participant; if it was less than 20 kg/m^2^, the participant was judged to have evidence of WL even if no WL windows were detected.

### 2.3 WGS Analysis

WGS of participant white blood cells, and its quality control, were performed separately and have previously been reported for both the All of Us study [15] and the TOPMed Initiative [16]. In brief, both studies used Illumina next‐generation sequencing instruments (San Diego, CA) to conduct sequencing to a read‐depth of 30× or greater and then subjected both samples and variants to extensive quality control. The curated sequencing data were made available to researchers using the hg38 reference genome assembly, and in reporting our results, we use the hg38 build numbering convention.

In studies from the TOPMed Initiative, single variant (SV) analyses were performed on freeze 9b data using the GENESIS package [18] as implemented in the BioData Catalyst cloud computing environment [19], and associations between variants with a minor allele count (MAC) greater than 20 and the presence of our WL phenotype were corrected for age, sex and the first five principal components of genetic ancestry. Among TOPMed studies, COPDGene and SPIROMICS were separated on the basis of self‐identified race into NHW and Black/African–American (B/AA) cohorts, which were analysed separately. Other TOPMed studies were either too racially homogenous (FHS, JHS, and ECLIPSE) or contained too few participants with COPD to permit stratification (MESA and CHS) and so were analysed as one group. A final cosmopolitan analysis of all TOPMed participants in selected studies with available data was run without respect to self‐identified race. In the All of Us study, SV analyses were performed on version 7 genomic reads using SAIGE [20], and associations between all variants with a MAC > 40 and the presence of our WL phenotype were corrected for age, sex and the first five principal components of genetic ancestry. A cosmopolitan analysis of all participants, as well as race‐stratified analyses of participants identifying as NHW or B/AA, was performed in All of Us. Statistical significance for single‐variant analyses was set at 5 × 10^−8^. Results were visualized using quantile–quantile (QQ) and Manhattan plots.

### 2.4 Meta‐Analysis of Single Variant Results

**FIGURE 1:**
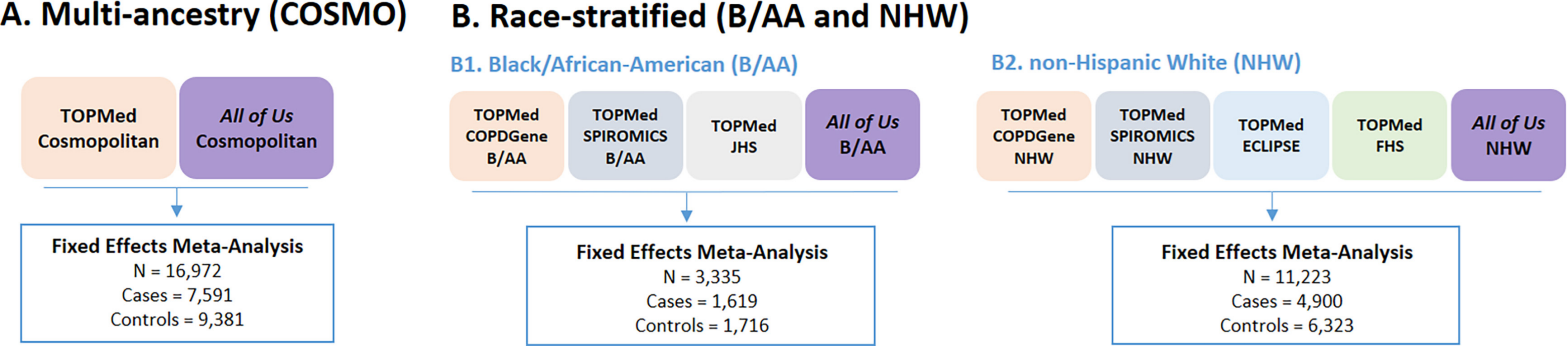
Design of single variant meta‐analyses of weight loss in COPD. Abbreviations: B/AA, Black/African–American; CHS, Cardiovascular Health Study; COPDGene, Genetic Epidemiology of COPD; ECLIPSE, Evaluation of COPD to Longitudinally Identify Predictive Surrogate Endpoints; FHS, Framingham Heart Study; JHS, Jackson Heart Study; MESA, Multi‐Ethnic Study of Atherosclerosis; NHW, non‐Hispanic white; SPIROMICS, SubPopulations and InteRmediate Outcome Measures in COPD Study; TOPMed, Trans‐Omics for Precision Medicine Initiative.

Three single variant meta‐analyses (Figure 1) were conducted using the METAL fixed‐effects meta‐analysis software on the following populations: TOPMed and All of Us Cosmopolitan Analyses (the COSMO meta‐analysis), all B/AA‐predominant cohorts (COPDGene B/AA, SPIROMICS B/AA, JHS and All of Us B/AA; the B/AA meta‐analysis) and all NHW‐predominant cohorts (COPDGene NHW, SPIROMICS NHW, ECLIPSE, FHS and All of Us NHW; the NHW meta‐analysis). Before meta‐analysis, all multivalent variants were removed and only variants that appeared in at least two cohorts in any given meta‐analysis were reported. Each variant was tested for heterogeneity of effect across cohorts. Meta‐analysis results were visualized using QQ and Manhattan plots.

### 2.5 Gene‐Based Tests and Meta‐Analysis

Gene‐based analyses were also performed in both TOPMed freeze 9b and All of Us version 7 with SAIGE‐GENE+, which combines kernel‐based and burden‐based testing to produce an overall test of association at each gene [21]. Two sets of variants were selected for these tests using the freeze 8 TOPMed Annotation Explorer in BioData Catalyst [22]: one composed only of high‐confidence loss‐of‐function variants as identified by LOFTEE [23], designated ‘LoF’, and the other consisting of the loss‐of‐function variants as well as missense, insertion–deletion and noncoding variants predicted to be deleterious by a SIFT4G score < 0.05 [24], a PolyPhen‐2 HDIV or HVAR score > 0.5 [25], a FATHMM‐XF score > 0.5 [26], or their location within an enhancer or promoter region designated as ‘LoF+’. These annotation groups were adopted from previously proposed annotation sets in the TOPMed population [27]. Given the relatively low numbers of participants involved in this rare variant analysis, we focused our analysis on the most permissive minor allele fraction cut‐off (0.01) and the broadest annotation set, ‘LoF+’. We set our threshold for significance by applying a Bonferroni correction for 20 000 independent gene tests to an *α* of 0.05, resulting in a significance threshold of 2.5 × 10^−6^. Cosmopolitan gene‐based analyses, as well as race‐stratified analyses using the two largest racial populations (B/AA and NHW), were conducted separately in TOPMed and All of Us. Gene‐based *p*‐values from TOPMed and All of Us were combined using Fisher's method [28], maintaining the same Bonferroni‐corrected *p*‐value for significance. Results were visualized using QQ and Manhattan plots.

### 2.6 Fine‐Mapping Using PAINTOR and LocusZoom

Variants associated with WL in COPD at a level of genome‐wide significance (*p* < 5 × 10^−8^), either in single cohorts or in overall meta‐analyses, were further characterized by fine‐mapping with PAINTOR [29]. The enumerate function was set to either 1 or 2 for each locus, with the most parsimonious set of credible set results presented in this manuscript. For fine‐mapping, we used a LD panel from TOPMed participants used in the cosmopolitan, NHW or AA analysis to correspond to the population of the original analysis. Regional association plots for these loci were composed with LocusZoom [30].

### 2.7 In Silico Transcriptomics Analysis

To simulate the effects results from our single variant meta‐analysis results on tissue‐specific levels of genetically regulated gene expression, we used MetaXcan software [31] and tissue‐specific expression quantitative trait locus data from the GTEx Consortium [32]. A gene‐level significance threshold of 2.5 × 10^−6^ was considered statistically significant, and we performed testing using results from the B/AA, Cosmo and NHW meta‐analyses. Given the pathophysiologic processes implicated in WL in COPD, we focused this analysis on predicted transcriptomic changes in skeletal muscle, subcutaneous adipose tissue, visceral omental adipose tissue and the lung. Nominally significant MetaXcan results (*p* < 10^−4^) were investigated using gene set enrichment analysis.

### 2.8 Look‐Up of Results From Prior WL in COPD GWAS

We compared significant results from our prior GWAS of WL in COPD [9] with results from the current analyses. We directly compared single variant hits from the prior analysis and used LocusZoom to assemble regional association plots of prior significant gene‐level associations.

### 2.9 Colocalization With Prior GWAS of Related Traits

We utilized the GWAS catalogue [33] to find genes at loci discovered as part of this analysis that had previously been implicated for the related phenotypes body mass index, FEV_1_, COPD, frailty, sarcopenia and WL after bariatric surgery.

## 3 Results

### 3.1 TOPMed Participants With COPD Show High WL Rates and Cumulative Smoke Exposure

Participant characteristics are presented in Table 1. Briefly, participants with COPD in TOPMed studies ranged in average age from 63–79 years at last study visit. Racial breakdowns varied by study recruitment strategy: While FHS and JHS were composed of all NHW and B/AA individuals, respectively, and ECLIPSE had a significant majority of NHW participants, the other studies (CHS, COPDGene, MESA, and SPIROMICS) had higher levels of racial and ethnic diversity. Mean cigarette‐smoking exposure, measured as packs per day times years of smoking exposure, ranged from 36 to 53 pack‐years, with FHS showing the lowest and SPIROMICS the highest mean smoke exposure. Incidence of WL or low BMI in participants with COPD ranged from 31% in ECLIPSE to 54% in FHS.

**TABLE 1:** Participant characteristics by study.

	FHS	MESA	CHS	JHS	COPDGene	ECLIPSE	SPIROMICS	All of Us
Participants	546	452	795	104	3097	1888	1234	8856
Male sex	315 (58)	309 (68)	486 (61)	54 (52)	1642 (53)	1238 (66)	701 (57)	4757 (54)
Age (mean ± SD)	72.8 ± 8.0	72.8 ± 8.8	79.2 ± 5.5	68.2 ± 11	68.8 ± 8.7	63.1 ± 7.2	67.5 ± 8.2	65.9 ± 9.9
Race: non‐Hispanic white	546 (100)	244 (54)	689 (87)	0 (0)	2311 (75)	1845(98)	995 (81)	5483 (62)
Race: Asian	0 (0)	37 (8.2)	0 (0)	0 (0)	0 (0)	0 (0)	12 (1.0)	54 (0.6)
Race: non‐Hispanic Black/AA	0 (0)	118 (26)	102 (13)	104 (100)	786 (25)	29 (1.5)	167 (14)	2278 (26)
Race: Hispanic	0 (0)	53 (12)	0 (0)	0 (0)	0 (0)	0 (0)	42 (3.4)	0 (0)^a^
Race: Native/other	0 (0)	0 (0)	4 (0.5)	0 (0)	0 (0)	0 (0)	17 (1.4)	944 (11)^a^
Pack‐years (mean ± SD)	36.3 ± 24	39.6 ± 30	43.6 ± 27	32.4 ± 30^b^	50.3 ± 26	48.7 ± 27	52.9 ± 26	33.2 ± 28
Weight loss	295 (54)	160 (35)	368 (46)	45 (43)	1457 (47)	590 (31)	389 (32)	4287 (48)

*Note:* All data presented as number (%) except where noted.

Abbreviations: AA, African–American; CHS, Cardiovascular Health Study; COPDGene, Genetic Epidemiology of COPD; ECLIPSE, Evaluation of COPD to Longitudinally Identify Predictive Surrogate Endpoints; FHS, Framingham Heart Study; JHS, Jackson Heart Study; MESA, Multi‐Ethnic Study of Atherosclerosis; SD, standard deviation; SPIROMICS, SubPopulations and InteRmediate Outcome Measures in COPD Study.

^a^ All of Us does not classify Hispanic as a race and does not permit reporting on groups with less than 20 participants.

^b^ J.H.S. does not have smoke exposure history for three participants.

### 3.2 All of Us Participants With COPD Also Show High WL Rates

Participants with COPD from the All of Us study were generally of similar age to TOPMed participants (66 vs. 63–79 years; Table 1), were slightly less likely to be NHW compared to TOPMed cohorts (62% vs. 54%–100%), showed amounts of smoke exposure that were similar to community‐based TOPMed studies (33 vs. 32–43 average pack years) and experienced similar overall rates of WL or low BMI (48% vs. 31%–54%).

### 3.3 Single Variants on Chromosomes 3 and 5 Are Associated With WL in Individual Cohort Analyses

Two variants were statistically significantly associated with WL in COPD in the All of Us study (Table 2). These variants were rare with a large effect size. The first, a rare variant intergenic to *PPP4R2* and *PDZRN3*, with AAF of 0.004 on chromosome 3 (chr3: 73345901:A:G), was associated with WL in COPD among NHWs with an odds ratio (OR) = 0.18 and a 95% confidence interval (CI) = 0.10–0.33, *p* = 9.44 × 10^−9^. This result was similar in the cosmopolitan analysis in All of Us (AAF = 0.003, OR = 0.21, 95% CI = 0.12–0.35, *p* = 8.84 × 10^−9^), which comprised all 8856 participants with COPD. This association did not replicate in the TOPMed Cosmopolitan analysis (AAF = 0.003, OR = 1.03, *p* = 0.91). The second variant, found in participants of Black or African–American race (chromosome 5, position 45 271 359:TACACAC:T, intronic to the *HCN* gene), was significantly associated with WL or low BMI in All of Us (AAF = 0.037, OR = 2.43, 95% CI = 1.78–3.31, *p* = 1.95 × 10^−8^) but was excluded from analysis in the TOPMed cohorts due to failing variant quality control. Manhattan and Q–Q plots for each of the study‐ and race‐stratified analyses, as well as the cosmopolitan analyses within TOPMed and All of Us, can be found in the Supporting Information appendix (Figures [Link], [Link], [Link], [Link], [Link], [Link], [Link], [Link], [Link], [Link], [Link], [Link], [Link]) and generally indicate no evidence of significant genomic inflation.

**TABLE 2:** Single variants associated with WL in COPD in single cohorts.

Study	Race	Chr:Position	Ref	Alt	Total N	AAF	OR	95% CI	*p*	Gene	Annotation
AoU	B/AA	5:45271359	TACACAC	T	2278	0.037	2.43	1.78–3.31	1.95E‐08	*HCN1*	Intronic
AoU	NHW	3:73345901	A	G	5483	0.004	0.18	0.10–0.33	9.44E‐09	*PPP4R2, PDZRN3*	Intergenic
AoU	COSMO	3:73345901	A	G	8856	0.003	0.21	0.12–0.35	8.84E‐09	*PPP4R2, PDZRN3*	Intergenic

Abbreviations: AAF, alternate allele fraction; Alt, alternate allele; AoU, All of Us Research Program; B/AA, Black/African–American; Chr, chromosome; CI, confidence interval; COSMO, cosmopolitan analysis; NHW, non‐Hispanic white; OR, odds ratio; Ref, reference allele.

### 3.4 Two Novel Loci Significantly Associated With WL in COPD

**FIGURE 2:**
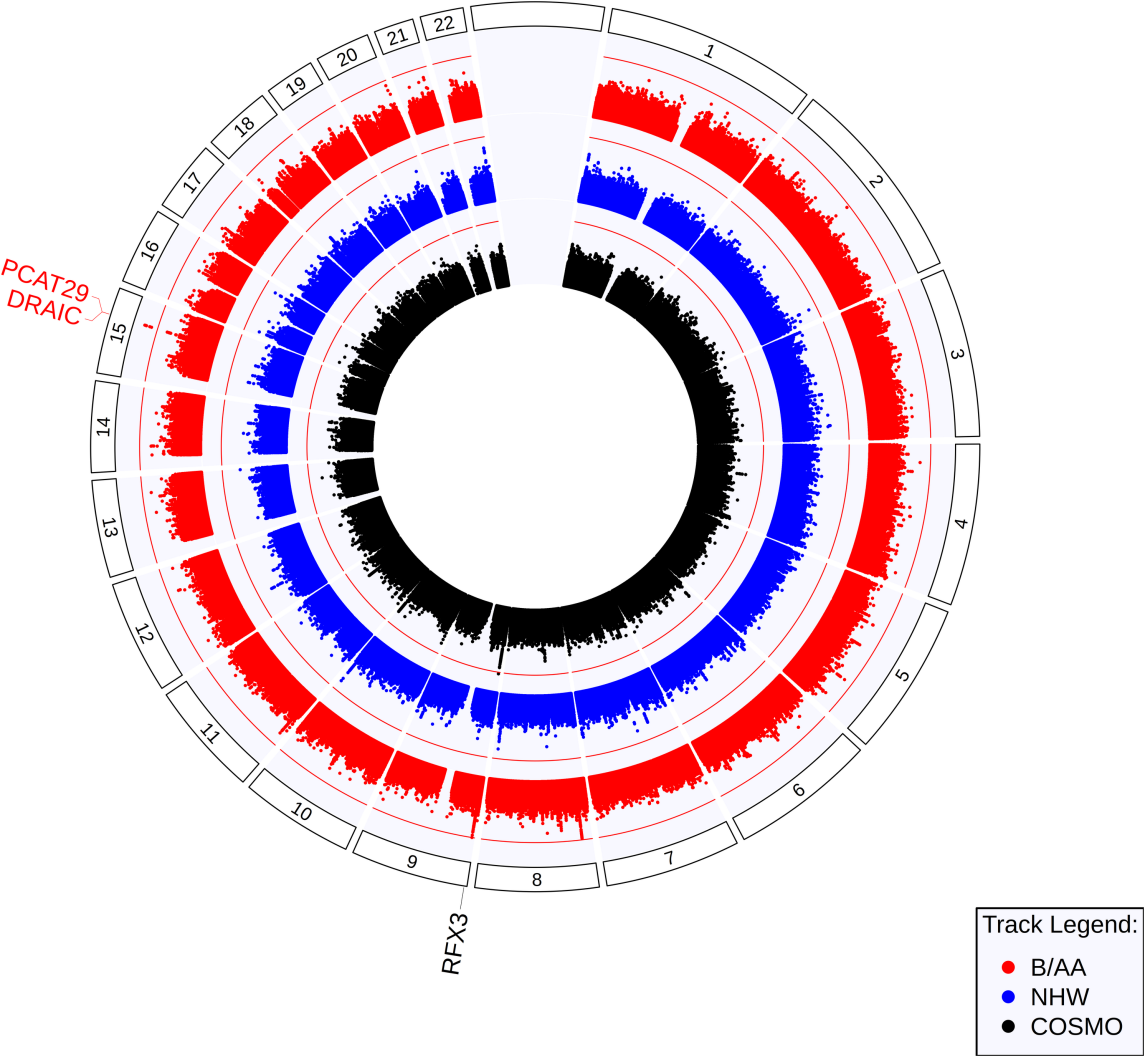
Results of single variant meta‐analyses of weight loss in COPD. This circos plot shows meta‐analysed single‐variant results from the B/AA (3335 Black and African–American participants, displayed on the red, outer track), NHW (11 223 non‐Hispanic white participants, displayed on the second, blue track) and COSMO (all 16 972 participants, analysed together by cohort, displayed on the inner, black track) designs. Genome‐wide significant results are identified by the nearest genes in the colour of the track on which the variants are found. The outer ring of white boxes corresponds to the chromosome on which those variants are found.

Our meta‐analyses demonstrated significant associations between WL or low BMI and variants in two loci (Table 3 and Figure 2): on chromosome 15 within the *DRAIC* gene in our meta‐analysis of cohorts of Black or African–American participants (the B/AA analysis, Table S1) and on chromosome 9 within the *RFX3* gene in the meta‐analysis of the cosmopolitan analyses of the TOPMed and All of Us cohorts (the COSMO analysis, Table S2). The lead variant from the locus on chromosome 15 (chr15:69571341 A:G, AAF = 0.6113–0.6617, OR = 1.37, 95% CI = 1.23–1.51, *p* = 1.29 × 10^−9^) is exonic within a long noncoding RNA (lncRNA) gene and is associated with consistently positive effects on odds of WL or low BMI across all four cohorts. The two significant variants in the COSMO meta‐analysis (chr9:3390983:T:C and chr9:3391066:T:C, AAF = 0.97–0.98, OR = 1.50, 95% CI = 1.31–1.73, *p* = 1.06 × 10^−8^) are located within an intron of the *RFX3* gene. Of note, while found in the COSMO meta‐analysis, this allele was largely present only in participants of Black/African–American race (89.7% and 97.2% of all minor alleles at this locus from B/AA participants in All of Us and TOPMed, respectively). The meta‐analysis of the cohorts of NHW participants (Table S3) did not demonstrate any associations at our genome‐wide significance threshold.

**TABLE 3:** Single variants significantly associated with WL in COPD In meta‐analyses.

Group	Chr:Position	Ref	Alt	Total N	Min Freq	Max Freq	OR	95% CI	*p*	Direction^a^	Closest Gene(s)	Annotation	HetISq	HetPVal
B/AA	15:69571341	A	G	3335	0.611	0.662	1.37	1.23–1.51	1.29E‐09	++++	*DRAIC*	ncRNA_exonic	0.0	0.69
B/AA	15:69570512	A	G	3335	0.335	0.387	0.73	0.66–0.81	1.79E‐09	−−−−	*DRAIC*	ncRNA_intronic	0.0	0.74
B/AA	15:69570426	T	C	3335	0.335	0.387	0.73	0.66–0.81	2.07E‐09	−−−−	*DRAIC*	ncRNA_intronic	0.0	0.74
B/AA	15:69574857	G	GA	3335	0.344	0.391	0.74	0.67–0.82	5.41E‐09	−+−	*DRAIC,PCAT29*	Intergenic	0.0	0.62
B/AA	15:69576512	A	G	3335	0.608	0.656	1.34	1.21–1.48	8.94E‐09	++−+	*DRAIC,PCAT29*	Intergenic	0.0	0.61
COSMO	9:3390983	T	C	16 972	0.967	0.981	1.50	1.31–1.73	1.06E‐08	++	*RFX3*	Intronic	0.0	0.70
COSMO	9:3391066	T	C	16 972	0.967	0.981	1.50	1.31–1.73	1.06E‐08	++	*RFX3*	Intronic	0.0	0.70

Abbreviations: Alt, alternative allele; B/AA, Black and African–American meta‐analysis; Chr, chromosome; COSMO, Cosmopolitan meta‐analysis; HetISq, heterogeneity I‐squared value; HetPVal, heterogeneity *p*‐value; Max Freq, maximum alternative allele frequency; Min Freq, minimum alternative allele frequency; OR, odds ratio; Ref, reference allele; SE, standard error.

^a^ Denotes the direction of the estimated effect in each study, in order as depicted in Figure 1.

### 3.5 Fine‐Mapping of Variants Associated With WL in COPD Identifies Possible Effect Variants

**FIGURE 3:**
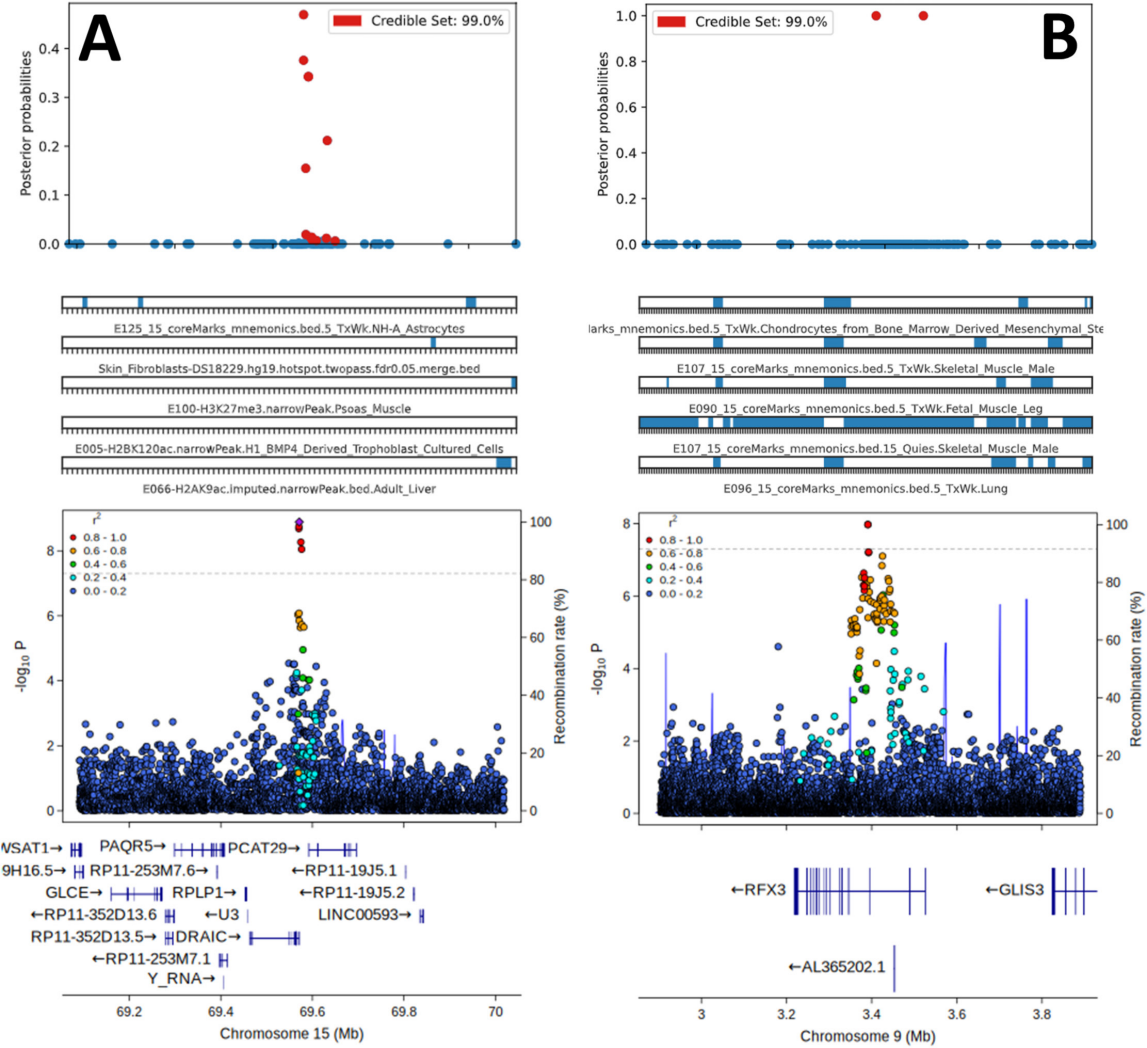
Fine‐mapping plots of genome‐wide significant meta‐analysed single variants associated with weight loss in COPD. Upper panels show posterior probabilities of lying within a credible set, the middle panel shows the annotation tracks used in PAINTOR analysis, while the lower panels show LocusZoom regional association plots. (A) The chromosome 15 locus within *DRAIC* from the meta‐analysis of B/AA meta‐analysis of populations of Black or African–American participants (B) The chromosome 9 locus within *RFX3* from the COSMO meta‐analysis of all participants from each cohort.

Within the locus on chromosome 15 from the B/AA meta‐analysis, the variant showing the highest posterior probability of membership in the predicted credible set of effect variants is the second leading variant from the meta‐analysis results (chr15:69570512:G:A, post_Cred_ [posterior probability of credible set membership] = 0.47, Figure 3A). Meanwhile, at the locus on chromosome 9 from the COSMO meta‐analysis, we identified two variants downstream of the discovery variants as being likely causative (chr9:3481864:TA:T and chr9:3381344:G:A, post_Cred_ = 1 for both, Figure 3B). Fine‐mapping of single‐cohort significant loci is shown in the Supporting Information (Figure S14): The chromosome 5 locus shows relatively little confidence in credible set generation, while the plots from the chromosome 3 locus show singleton variants without apparent LD with surrounding variants.

### 3.6 Rare Variants in RP11‐1O2.1 and RNU6‐565P Are Associated With WL in COPD

Plots of gene‐based meta‐analysis results (Figures [Link], [Link], [Link]) and single‐cohort results (Figures [Link], [Link], [Link], [Link], [Link], [Link]) can be found in the Supporting Information. The Q‐Q plots from these analyses generally show deflation of results as compared to expectations under the null. Despite this, two genes were significantly associated with WL or low BMI in our gene‐based tests, both within the All of Us study. Within the Black and African–American group, the uncharacterized RNA locus *RP11‐1O2.1* was associated with WL or low BMI (MAC = 61, *p* = 1.85 × 10^−6^; Table 4 and Figure S18), but this association did not remain significant after combination with results from Black/African–American participants in TOPMed (*p* = 2.63 × 10^−5^; Table S4, Figure S15). In the NHW cohort, rare variants within the pseudogene *RNU6‐565P* were found to be associated with increased risk of WL or low BMI (MAC = 46, *p* = 2.83 × 10^−^7; Table 4 and Figure S19). Despite only a single minor allele associated with this gene being identified within the TOPMed NHW cohort, this relationship showed a consistent direction of effect in All of Us and remained significant after *p*‐value combination (p_combined_ = 1.23 × 10^−6^, direction of effect positive in both studies; Tables 5 and S4 and Figure S16).

**TABLE 4:** Genes significantly associated with WL in COPD on rare variant analysis.

Study	Cohort	Chr	Gene	Ensembl ID	MAC	*p*	Burden *p*	Burden OR	Burden SE
AoU	NHW	7	*RNU6‐565P*	ENSG00000222869	46	2.83E‐07	4.92E‐07	1.069	0.013
AoU	B/AA	18	*RP11‐1O2.1*	ENSG00000263547	61	1.85E‐06	7.24E‐07	0.944	0.012

Abbreviations: AoU, All of Us; B/AA, Black/African–American; Burden OR, odds ratio from burden test; Burden SE, standard error from burden test; Chr, chromosome; MAC, minor allele count; NHW, non‐Hispanic white.

**TABLE 5:** Genes significantly associated with WL in COPD on rare variant analysis after *p*‐value combination.

Gene information						TOPMed		All of Us		Combined analysis	
Race	Chr	Start	End	Gene	Ensembl ID	MAC	*p*	MAC	*p*	Direction	*p*
NHW	7	37 351 143	37 351 242	*RNU6‐565P*	ENSG00000222869	1	0.25	46	2.83E‐07	++	1.23E‐06

Abbreviations: AoU, All of Us; Burden OR, odds ratio from burden test; Burden SE, standard error from burden test; Chr, chromosome; MAC, minor allele count; NHW, non‐Hispanic white; TOPMed, Trans‐Omics for Precision Medicine Initiative.

### 3.7 MetaXcan Predicts Differential Genetically Regulated Gene Expression in LINC00959 in Visceral Adipose Tissue

Across the three meta‐analysis designs and four tissues, genetically regulated gene expression of one transcript was significantly associated with WL in COPD: *LINC00959* in visceral omental adipose tissue (*p* = 1.16 × 10^−6^ in the COSMO meta‐analysis; Table S5 and Figure S25). Differences in genetically regulated gene expression were predicted in a total of 21 genes at a *p*‐value of 10^−4^ or less across all analyses (Table S5 and Figures [Link], [Link], [Link]), but no gene sets were significantly enriched in these genes.

### 3.8 Prior WL in COPD GWAS Results Were Not Replicated

Our prior analysis [9] found a genome‐wide significant signal at the rs35368512 variant (chr4:43230650:C:T in hg38) in Black/African–American participants from COPDGene. This variant is no longer significant at the genome‐wide level in our analysis of that population (*p* = 6.5 × 10^−4^). Notably, more participants are included in this population in the current analysis (*n* = 786) as compared to our prior GWAS (*n* = 401). Additionally, though *EFNA2* and *BAIAP2* showed evidence of gene‐level association in Black/African–American and NHW participants, respectively, in our prior analysis, there are no significant signals of regional association in our analysis of the same populations (Figure S27).

### 3.9 Colocalization of Genetic Association Signals With BMI, Frailty, and FEV1

None of the single variants identified in this analysis have had prior associations with related traits. At the gene level, both *DRAIC* and *PDZRN3* had two prior associations with related traits: variants within and near *DRAIC* have been associated with BMI [34] and frailty [35], while variants within and near *PDZRN3* have been associated with BMI [34, 36] and FEV_1_ [37]. Variants within or near *RFX3* [38] and *LINC00959* (also known as *C10orf143*) [36] were both previously associated with BMI as well. No loci previously associated with COPD, sarcopenia, lean body mass or WL were associated with WL in COPD in the current study at a level reaching genome‐wide significance. Variants within and near *HCN1*, *LOC339298* and *RNU6‐565P* did not have any prior genetic associations with traits of interest.

## 4 Discussion

We identified four novel loci associated with WL in people with COPD in our single‐variant analysis, including two loci that are robust across study populations and WL definitions. We further identified two genes where the presence of rare variants was associated with risk for WL or low BMI in COPD. Finally, our genetic analysis results, when integrated with known eQTLs, predict differential genetically regulated expression of one gene in visceral fatty tissues. Taken together, this work expands our understanding of the genetic risks for WL and low BMI in people with COPD and provides insights that may guide further mechanistic studies.

Among the four novel loci associated with WL in COPD, one of the most intriguing is the locus within the *DRAIC* gene. The presence of prior associations between genetic variants in *DRAIC* with BMI and frailty is interesting, but this locus is of specific interest because *DRAIC* is a long noncoding RNA (lncRNA) that inactivates IκB kinase (IKK), reducing NF‐κB activity [39]. While IKK and downstream NF‐κB activation have long been implicated in the pathogenesis of muscle wasting in cachexia [40], no one has previously linked genetic variants within a lncRNA modulator of this pathway to WL in COPD. The lead variant in our genomic and fine‐mapping analyses is exonic, but it is located in the latter portion of the 5th exon, which occurs after the region of bases 701–905 situated in exon 4 that is most strongly associated with binding of IKK subunits [39]. This may imply that this variant may not be associated with the direct binding of target molecules, but the affected area of the lncRNA could certainly be structural or, alternatively, one of the intronic variants in high LD with the lead variant may affect splicing effectiveness or transcript stability; further mechanistic study will be required to determine the way that these genetic variants modulate risk for WL in COPD. The modulation of IKK and NF‐κB by peptide or small molecule inhibitors has been previously studied with promising effects in mouse models of cancer cachexia [41], but the development of lncRNA therapeutics remains in its infancy [42]. Future studies of therapeutic uses of lncRNAs, such as *DRAIC*, could prove useful in the search for effective treatments of wasting syndromes in COPD.

Our second locus with consistent effect size and direction across multiple study populations was located within the *RFX3* gene, which we found has also been previously associated with BMI. The two variants at this locus appear to be predominantly found in Black/African–American participants, despite its discovery in the COSMO meta‐analysis. All of Us has significantly more participants who racially identify as ‘Other’ and who are included in the COSMO analysis. This finding in particular highlights the importance of performing Cosmopolitan analyses to identify variants which otherwise could be overlooked using other approaches. The association between WL and genetic variants in *RFX3* is intriguing because *RFX3* is a transcription factor highly associated with ciliated cell differentiation within the lung and other tissues [43] but is not known to be highly active in the skeletal muscle of adults. This raises the possibility of cross‐talk between tissues affected by a certain genetic variant and a distal effect tissue, such as skeletal muscle or adipose tissue. There is some prior research that could lend credence to this possibility, as mutations affecting posttranslational acylation of the RFX3 protein are associated with alterations in Hedgehog signalling [44], and constitutive Hedgehog activation within bone has previously been associated with diffuse muscle atrophy [45]. This suggests that genetic variants in the *RFX3* gene, in concert with or in reaction to the global ciliopathy frequently found in people with COPD [43], could plausibly affect skeletal muscle tissue bulk via systemic effects of signalling through the Hedgehog pathway. This presents a possible link between the lung pathophysiology of COPD and the systemic wasting diseases associated with it and bears further investigation.

Our third locus, consisting of a single rare variant on chromosome 3 in the NHW and cosmopolitan analyses from All of Us, sits in an intergenic region between genes *PPP4R2* and *PDZRN3*. Other variants near *PDZRN3* have previously shown associations with BMI [34, 36] and FEV_1_ [37], which gives some evidence to support the biologic plausibility of this finding. Unfortunately, this variant was not included in the NHW results from TOPMed cohorts due to its low allele frequency (*n* = 50 across all TOPMed studies) and our MAC cut‐off for each study (MAC < 20). Additionally, in the cosmopolitan meta‐analysis, there was evidence of effect size heterogeneity as results from TOPMed showed an opposite effect direction. The gene *PDZRN3* encodes PDZ domain containing RING‐finger family protein number 3, a ubiquitin ligase that acts to prevent apoptosis through maintaining levels of cyclin A2 in myoblasts [46]. Meanwhile, the *PPP4R2* gene encodes protein phosphatase 4 regulatory subunit 2, which has been suggested to be involved in DNA damage repair and acts to improve survival in diseased neurons [47]. Given the heterogeneous results associated with this locus, further study is necessary to clarify whether this is a spurious association or whether there is reproducible evidence of genetic association at this locus.

While statistically significant, our fourth locus included a single variant on chromosome 5 within the *HCN1* gene in Black/African–American All of Us participants and could not be compared to results from TOPMed studies due to removal during variant quality control in TOPMed. This variant is one of many at this multi‐allelic site that shows evidence of dinucleotide repeat expansion, but this specific variant is the only one that shows evidence of any association with WL in our analysis, further reducing our confidence in the value of this specific locus for further study. Our fine‐mapping results also underscore the low confidence we have in this result, as the PAINTOR algorithm has significant difficulties assembling a parsimonious credible set.

From our rare variant analysis, we identified rare variants within the *RNU6‐565P* and *RP11‐1O2.1* genes as being associated with WL. Both genes are thought to be noncoding RNAs, with *RNU6‐565P* identified as a pseudogene for the small nuclear RNA U6, a class of RNA molecules that participate in splicing of pre‐mRNAs. This is one of many *RNU6* pseudogenes, and there is little currently known about its function or significance, though other *RNU6* pseudogenes have been previously identified as being associated with acute response to skin infection with *Pseudomonas aeruginosa* [48]. The *RP11‐1O2.1* gene, also known as *LOC339298*, is close to a variant previously associated with FEV_1_/FVC ratios in never smokers [49], which suggests the possibility of pleiotropic genetic effects of this largely uncharacterized gene on both skeletal muscle and lung tissue.

In our genome‐predicted transcriptomic analysis using MetaXcan, variants across three of our meta‐analyses (ALL, COSMO and NHW) suggested altered expression levels of *LINC00959*, a gene also known as *C10orf143*, in visceral adipose tissue of the omentum. This gene has been previously characterized in a cohort of colorectal cancer patients, where higher expression was linked to improved survival and lower cancer stage [50] and has been shown to be down‐regulated in multiple cancer types [51]. This, in addition to prior evidence of genetic association between variants in *LINC00959* and BMI suggests that *LINC00959*, much like *DRAIC*, may be involved in cellular responses to pathologic processes and that further work to elucidate its mechanism of action is needed.

Some variants and regions of interest from our prior analysis [9], including *EFNA2* and *BAIAP2*, were no longer observed as significantly associated with WL in COPD in the current analysis. This may be due to multiple methodologic changes, including (1) the use of WGS data, (2) addition of more participants with longitudinal data in this analysis or (3) our switch from using participant‐reported unintentional WL in COPDGene to objectively measured WL or low BMI to better match other TOPMed studies where information about unintentional WL was not available. The use of data from additional participants (*n* = 786 vs *n* = 401 B/AA participants in the current and previous analyses, respectively) and re‐definition of our phenotype are likely the primary cause of the disparity of results between this study and our prior study. Despite these changes, the chromosome 4 variant previously identified in B/AA participants from COPDGene still shows an association with WL (*p* = 6.5 × 10^−4^), though it no longer reaches genome‐wide statistical significance. Thus, though we attempted to replicate our prior GWAS results, the results from our current study indicate that they were not fully robust to our changes in methodology.

In our analysis, it bears noting that we chose to stratify study populations on the basis of self‐identified race and ethnicity at the time of study enrollment, which is an imperfect substitute for genetic ancestry. This decision allowed us to account for social and environmental factors that may differ between racial groups while still accounting for genetic relatedness using linear mixed models as implemented in the SAIGE and GENESIS pipelines. We performed cosmopolitan analyses to counter any bias caused by racial stratification, which served to increase our power to detect associations between genetic variants at the *RFX3* locus and WL in COPD.

In this study, as in many other genomics studies [52], we found evidence of association at some variants that did not replicate across ancestral groups or across cohorts. A simplistic view of these results would say this underscores the importance of replicating genetic associations in other populations, as some of these associations may be false positives and may not hold any true biological significance. However, there are myriad other explanations of why heterogeneous genetic association signals between cohorts may be, in fact, biologically valid and advance our understanding of disease aetiology. The use of different ascertainment criteria across cohorts ensures a certain amount of phenotypic heterogeneity between cohorts. This is further superimposed on the natural heterogeneity of the causes of WL in people with COPD, which may be distributed unevenly between groups. In addition, differences in recruitment processes and environmental exposures across cohorts may result in unique environmental risks or gene–environment interactions that are private to a given cohort. Different timeframes for ascertainment mean that episodes of WL closely linked to death may be missed by cohorts with visits spaced further apart, depleting these cohorts of power to detect specific biologic effects. This emphasizes the need for continued genomic analysis to better understand the true nature of results that vary across cohorts and ancestral groups.

This WGS analysis has many strengths, including a relatively diverse participant population, both in terms of race and ethnicity, as well as our utilization of both general, population‐based and smoking‐enriched cohorts. This diversity is crucial as it allows for the study of loci that may only be present in certain populations, such as the Black/African–American population, which is the primary source of the loci discovered on meta‐analysis. Prior genomic studies have frequently been predominantly conducted in NHW participants, and our results underscore the importance of recruiting and analysing diverse populations. The robust effect direction and effect size of the single variants identified by meta‐analysis also speak to the generalizability and face validity of our methods. We used more finely detailed WGS data, more advanced analytical strategies employing the use of generalized linear mixed models and larger study sizes compared to our prior analyses. It is possible that some of these results were found to be significant originally due to issues related to sample size. Our current analysis shows greater robustness across studies and ancestries and underscores the importance of revisiting prior results as new techniques and information become available.

Conversely, this study did have some limitations. The included studies had differing levels of data granularity, which necessitated heterogeneous phenotype definitions that may reduce our power to detect significant genetic associations. In addition, some included studies had low numbers of participants with COPD and thus had reduced power to detect lower frequency variants. The use of different genomic software across cloud computing environments was another possible source of heterogeneity due to differences in model methodology. Our inclusion of smoking history in our COPD phenotype definition may limit generalizability to people with COPD from non‐cigarette exposures.

In conclusion, we have identified four novel genetic risk loci and three genes of interest that are associated with the presence of WL or low BMI in research participants with COPD. These results represent new molecular insights into the pathophysiology of wasting disorders in people living with COPD and highlight potential areas for validation studies and/or further mechanistic research.

## Ethics Statement

All human studies referenced above were approved by the local Institutional Review Board and have been performed in accordance with the Declaration of Helsinki and its amendments. All participants provided informed consent prior to inclusion in this study, and care has been taken to reduce the likelihood of any details that might disclose participant identities.

## Conflicts of Interest

The authors declare no conflicts of interest.

## Supporting information


**Data S1:** Supplementary information.

**Table S1:** Top genetic risk variants from a study‐stratified fixed effects meta‐analysis in Black/African–American participants for weight loss in COPD. Results are sorted from smallest *p*‐value to largest. Variants with HetPVal < 0.05 are highlighted in red, indicating heterogeneity in estimated effect. Abbreviations: Alt, alternate allele; Alt Freq, alternate allele frequency; B/AA, Black/African–American ancestry; Chr, chromosome; COPDGene, Genetic Epidemiology of COPD; HetChiSq, heterogeneity chi‐squared statistic; HetDf, heterogeneity degrees of freedon; HetISq, heterogeneity I‐squared value; OR, odds ratio; Pos, position; Ref, reference allele; SPIROMICS, SubPopulations and InteRmediate Outcome Measures in COPD Study; StdErr, standard error.
**Table S2:** Top genetic risk variants from a cosmopolitan fixed effects meta‐analysis in all participants for weight loss in COPD. Results are sorted from smallest *p*‐value to largest. Variants with HetPVal < 0.05 are highlighted in red, indicating heterogeneity in estimated effect. Abbreviations: Alt, alternate allele; Alt Freq, alternate allele frequency; Chr, chromosome; HetChiSq, heterogeneity chi‐squared statistic; HetDf, heterogeneity degrees of freedon; HetISq, heterogeneity I‐squared value; OR, odds ratio; Pos, position; Ref, reference allele; StdErr, standard error; TOPMed, Trans‐Omics for Precision Medicine Initiative.
**Table S3:** Top genetic risk variants from an ancestry‐ and study‐stratified fixed effects meta‐analysis in non‐Hispanic white participants for weight loss in COPD. Results are sorted from smallest p‐value to largest. Variants with HetPVal < 0.05 are highlighted in red, indicating heterogeneity in estimated effect. Abbreviations: Alt, alternate allele; Alt Freq, alternate allele frequency; Chr, chromosome; COPDGene, Genetic Epidemiology of COPD; ECLIPSE, Evaluation of COPD to Longitudinally Identify Predictive Surrogate Endpoints; HetChiSq, heterogeneity chi‐squared statistic; HetDf, heterogeneity degrees of freedon; HetISq, heterogeneity I‐squared value; NHW‐ non‐Hispanic white ancestry; OR, odds ratio; Pos, position; Ref, reference allele; SPIROMICS, SubPopulations and InteRmediate Outcome Measures in COPD Study; StdErr, standard error.
**Table S4:** Top genes from rare variant aggegrate association analysis of weight loss in COPD. Combined p‐values are sorted from the smallest to largest. Odds ratios and standard errors are based on a burden model. Genes without official HUGO Gene Nomenclature Committee names are denoted by ‘‐‐’. Abbreviations; B/AA, Black/African–American ancestry; Chr, chromosome; Comb. P‐value, p‐value from Fisher's method of p‐value combination; COSMO, Cosmopolitan analysis of all participants; ID, identification number; NHW, non‐Hispanic white ancestry; OR, odds ratio; SE, standard error; TOPMed, Trans‐Omics for Precision Medicine Initiative.
**Table S5:** Top MetaXcan‐predicted transcriptomic effects of meta‐analysed single variant results in four tissue types. Abbreviations: ALL, meta‐analysis of all cohort‐ and ancestry‐stratified cohorts; B/AA, meta‐analysis of cohorts enriched with Black and African–American participants; COSMO‐ meta‐analysis of cohorts containing all participants from TOPMed and All of Us Research Program; ID, identification number; NHW, meta‐analysis of cohorts enriched with non‐Hispanic white participants.

**Figure S1:** Single variant association testing for weight loss in Black/African–American (B/AA) participants with COPD in the All of Us Research Program. (A) Analysis design, including analysis method (SAIGE, Scalable and Accurate Implementation of GEneralized mixed model) and case/control counts. (B) Quantile–quantile plot of single variant results. (C) Manhattan plot of single variant results. Any genome‐wide significant results are identified with their position.

**Figure S2:** Single Variant Association Testing for Weight Loss in All Participants with COPD in the All of Us Research Program. (A) Analysis design, including analysis method (SAIGE—Scalable and Accurate Implementation of GEneralized mixed model) and case/control counts. (B) Quantile–quantile plot of single variant results. (C) Manhattan plot of single variant results. Any genome‐wide significant results are identified with their position.

**Figure S3:** Single Variant Association Testing for Weight Loss in non‐Hispanic white (NHW) Participants with COPD in the All of Us Research Program. (A) Analysis design, including analysis method (SAIGE—Scalable and Accurate Implementation of GEneralized mixed model) and case/control counts. (B) Quantile–quantile plot of single variant results. (C) Manhattan plot of single variant results. Any genome‐wide significant results are identified with their position.

**Figure S4:** Single variant association testing for weight loss in all participants with COPD in the Cardiovascular Health Study (CHS). (A) Analysis design, including analysis method (GENESIS—GENetic EStimation and Inference in Structured samples) and case/control counts. (B) Quantile–quantile plot of single variant results. (C) Manhattan plot of single variant results. Any genome‐wide significant results are identified with their position.

**Figure S5:** Single variant association testing for weight loss in Black/African–American (B/AA) participants with COPD in the genetic epidemiology of COPD (COPDGene) study. (A) Analysis design, including analysis method (GENESIS—GENetic EStimation and Inference in Structured samples) and case/control counts. (B) Quantile–quantile plot of single variant results. (C) Manhattan plot of single variant results. Any genome‐wide significant results are identified with their position.

**Figure S6:** Single variant association testing for weight loss in non‐Hispanic white (NHW) participants with COPD in the genetic epidemiology of COPD (COPDGene) study. (A) Analysis design, including analysis method (GENESIS—GENetic EStimation and Inference in Structured samples) and case/control counts. (B) Quantile–quantile plot of single variant results. (C) Manhattan plot of single variant results. Any genome‐wide significant results are identified with their position.

**Figure S7:** Single variant association testing for weight loss in all participants with COPD in the evaluation of COPD to longitudinally identify predictive surrogate endpoints (ECLIPSE) study. (A) Analysis design, including analysis method (GENESIS—GENetic EStimation and Inference in Structured samples) and case/control counts. (B) Quantile–quantile plot of single variant results. (C) Manhattan plot of single variant results. Any genome‐wide significant results are identified with their position.

**Figure S8:** Single variant association testing for weight loss in all participants with COPD in the Framingham Heart Study (FHS). (A) Analysis design, including analysis method (GENESIS—GENetic EStimation and Inference in Structured samples) and case/control counts. (B) Quantile–quantile plot of single variant results. (C) Manhattan plot of single variant results. Any genome‐wide significant results are identified with their position.

**Figure S9:** Single variant association testing for weight loss in all participants with COPD in the Jackson Heart Study (JHS). (A) Analysis design, including analysis method (GENESIS—GENetic EStimation and Inference in Structured samples) and case/control counts. (B) Quantile–quantile plot of single variant results. (C) Manhattan plot of single variant results. Any genome‐wide significant results are identified with their position.

**Figure S10:** Single variant association testing for weight loss in all participants with COPD in the multi‐ethnic study of atherosclerosis (MESA). (A) Analysis design, including analysis method (GENESIS—GENetic EStimation and Inference in Structured samples) and case/control counts. (B) Quantile–quantile plot of single variant results. (C) Manhattan plot of single variant results. Any genome‐wide significant results are identified with their position.

**Figure S11:** Single variant association testing for weight loss in Black/African–American (B/AA) participants with COPD in the SubPopulations and InteRmediate Outcome Measures in COPD Study (SPIROMICS) study. (A) Analysis design, including analysis method (GENESIS—GENetic EStimation and Inference in Structured samples) and case/control counts. (B) Quantile–quantile plot of single variant results. (C) Manhattan plot of single variant results. Any genome‐wide significant results are identified with their position.

**Figure S12:** Single variant association testing for weight loss in non‐Hispanic white (NHW) participants with COPD in the SubPopulations and InteRmediate Outcome Measures in COPD Study (SPIROMICS) study. (A) Analysis design, including analysis method (GENESIS—GENetic EStimation and Inference in Structured samples) and case/control counts. (B) Quantile–quantile plot of single variant results. (C) Manhattan plot of single variant results. Any genome‐wide significant results are identified with their position.

**Figure S13:** Single variant association testing for weight loss in all participants with COPD in the Trans‐Omics for Precision Medicine (TOPMed) Initiative. (A) Analysis design, including analysis method (GENESIS—GENetic EStimation and Inference in Structured samples) and case/control counts. (B) Quantile–quantile plot of single variant results. (C) Manhattan plot of single variant results. Any genome‐wide significant results are identified with their position.

**Figure S14:** Fine‐mapping results from single variant analyses of single populations. Credible set variants from PAINTOR (upper panel) and regional associated plots from LocusZoom (lower panel) of the chromosome 5 significant single variant in the *All of Us* Black/African–American cohort (A); the chromosome 3 significant single variant in the *All of Us* cosmopolitan cohort (B); and the chromosome 3 significant single variant in the *All of Us* non‐Hispanic white cohort (C). Annotation tracks used in the PAINTOR analysis are listed in the lower portion of the upper panel, while nearby protein‐coding genes are shown below the LocusZoom regional association plots.

**Figure S15:** Meta‐analysis of rare variant aggregate association testing results from Black/African–American (B/AA) participants from the Trans‐Omics for Precision Medicine (TOPMed) Initiative and *All of Us* Research Program. (A) Analysis design, including analysis method (SAIGE‐GENE, followed by Fisher's method of combining *p*‐values) and case/control counts. (B) Quantile–quantile plot of rare variant aggregate testing meta‐analysis results. (C) Manhattan plot of rare variant aggregate testing meta‐analysis results. Any genes meeting the genome‐wide significance threshold (*p* < 2.5 × 10^−6^) are identified with their gene name.

**Figure S16:** Meta‐analysis of rare variant aggregate association testing results all participants from the Trans‐Omics for Precision Medicine (TOPMed) initiative and *All of Us* Research Program. (A) Analysis design, including analysis method (SAIGE‐GENE, followed by Fisher's method of combining *p*‐values) and case/control counts. (B) Quantile–quantile plot of rare variant aggregate testing meta‐analysis results. (C) Manhattan plot of rare variant aggregate testing meta‐analysis results. Any genes meeting the genome‐wide significance threshold (*p* < 2.5 × 10^−6^) are identified with their gene name.

**Figure S17:** Meta‐analysis of rare variant aggregate association testing results from non‐Hispanic white (NHW) participants from the Trans‐Omics for Precision Medicine (TOPMed) Initiative and *All of Us* Research Program. (A) Analysis design, including analysis method (SAIGE‐GENE, followed by Fisher's method of combining *p*‐values) and case/control counts. (B) Quantile–quantile plot of rare variant aggregate testing meta‐analysis results. (C) Manhattan plot of rare variant aggregate testing meta‐analysis results. Any genes meeting the genome‐wide significance threshold (*p* < 2.5 × 10^−6^) are identified with their gene name.

**Figure S18:** Rare variant aggregate association testing results from Black/African–American (B/AA) participants from the *All of Us* research program. (A) Analysis design, including analysis method (SAIGE‐GENE) and case/control counts. (B) Quantile–quantile plot of rare variant aggregate testing meta‐analysis results. (C) Manhattan plot of rare variant aggregate testing meta‐analysis results. Any genes meeting the genome‐wide significance threshold (*p* < 2.5 × 10^−6^) are identified with their gene name.

**Figure S19:** Rare variant aggregate association testing results from all participants from the *All of Us* research program. (A) Analysis design, including analysis method (SAIGE‐GENE) and case/control counts. (B) Quantile–quantile plot of rare variant aggregate testing meta‐analysis results. (C) Manhattan plot of rare variant aggregate testing meta‐analysis results. Any genes meeting the genome‐wide significance threshold (*p* < 2.5 × 10^−6^) are identified with their gene name.

**Figure S20:** Rare variant aggregate association testing results from non‐Hispanic white (NHW) participants from the *All of Us* research program. (A) Analysis design, including analysis method (SAIGE‐GENE) and case/control counts. (B) Quantile–quantile plot of rare variant aggregate testing meta‐analysis results. (C) Manhattan plot of rare variant aggregate testing meta‐analysis results. Any genes meeting the genome‐wide significance threshold (*p* < 2.5 × 10^−6^) are identified with their gene name.

**Figure S21:** Rare variant aggregate association testing results from Black/African–American (B/AA) participants from the Trans‐Omics for Precision Medicine (TOPMed) Initiative. (A) Analysis design, including analysis method (SAIGE‐GENE) and case/control counts. (B) Quantile–quantile plot of rare variant aggregate testing meta‐analysis results. (C) Manhattan plot of rare variant aggregate testing meta‐analysis results. Any genes meeting the genome‐wide significance threshold (*p* < 2.5 × 10^−6^) are identified with their gene name.

**Figure S22:** Rare variant aggregate association testing results from all participants from the Trans‐Omics for Precision Medicine (TOPMed) Initiative. (A) Analysis design, including analysis method (SAIGE‐GENE) and case/control counts. (B) Quantile–quantile plot of rare variant aggregate testing meta‐analysis results. (C) Manhattan plot of rare variant aggregate testing meta‐analysis results. Any genes meeting the genome‐wide significance threshold (*p* < 2.5 × 10^−6^) are identified with their gene name.

**Figure S23:** Rare variant aggregate association testing results from non‐Hispanic White (NHW) participants from the Trans‐Omics for Precision Medicine (TOPMed) Initiative. (A) Analysis design, including analysis method (SAIGE‐GENE) and case/control counts. (B) Quantile–quantile plot of rare variant aggregate testing meta‐analysis results. (C) Manhattan plot of rare variant aggregate testing meta‐analysis results. Any genes meeting the genome‐wide significance threshold (*p* < 2.5 × 10^−6^) are identified with their gene name.

**Figure S24:** Predicted genetically regulated gene expression effects of meta‐analysed single variant associations with weight loss in Black/African–American (B/AA) participants with COPD from MetaXcan. Genes meeting nominal genome‐wide significance (*p* < 10^−5^) are identified by name.

**Figure S25:** Predicted genetically regulated gene expression effects of meta‐analysed single variant associations with weight loss in all (COSMO) participants with COPD from MetaXcan. Genes meeting nominal genome‐wide significance (*p* < 10^−5^) are identified by name.

**Figure S26:** Predicted genetically regulated gene expression effects of meta‐analysed single variant associations with weight loss in non‐Hispanic white (NHW) Participants with COPD from MetaXcan. Genes meeting nominal genome‐wide significance (*p* < 10^−5^) are identified by name.

**Figure S27:** Regional association plots of single variant associations with weight loss in COPD near previously implicated genes. Gene names are found along the bottom panel with exons represented by filled‐in boxes. *P*‐values are plotted on a negative log scale on the *y*‐axis, and each dot represents a variant.
