# Supplementary material for: Whole Genome Sequence Analysis of Weight Loss in 16 972 Participants With COPD Reveals Novel Risk Loci in DRAIC and RFX3
Source: J Cachexia Sarcopenia Muscle. 2026 Apr 23;17(3):e70293. doi: 10.1002/jcsm.70293 (PMC13106028; doi:10.1002/jcsm.70293)
Supplement: Supplementary file 1 — Data S1: Supplementary information. [file JCSM-17-e70293-s019.docx]

**SUPPLEMENTAL METHODS**

**Trans-Omics for Precision Medicine (TOPMed) Initiative Study Descriptions**

The Cardiovascular Health Study (CHS)^1^ recruited subjects 65 years or older to study risk factors of cardiovascular disease. The study collected phenotype data including information necessary to diagnose COPD and monitor changes in weight.

The Genetic Epidemiology of COPD (COPDGene) study^2^ is an observational study which enrolled COPD cases and control smokers at 21 U.S. clinical centers. Enrolled subjects are self-identified non-Hispanic white and non-Hispanic African-American subjects aged 45-80 with at least 10 pack-years of lifetime smoking history. Subjects with other diagnosed lung diseases except asthma and subjects with a first- or second-degree relative enrolled in the study were excluded. All enrolled subjects were invited to participate in two follow-up visits spaced roughly 5 years apart, as parts of Phase 2 and Phase 3 of COPDGene.

The Evaluation of COPD Longitudinally to Identify Predictive Surrogate Endpoints (ECLIPSE) trial^3^ (clinicalTrials.gov identifier: NCT00292552) is an international, observational study which enrolled subjects from 46 centers in 12 countries. Subjects were between the ages of 40-75 with ≥10 pack years of smoking who met spirometric criteria for chronic obstructive pulmonary disease (FEV1/FVC ratio < 0.7 and FEV1 < 80% predicted). ECLIPSE subjects were followed longitudinally every 6 months for three years.

The Framingham Heart Study (FHS)^4^ is the longest running, multi-generational observational population-based study. FHS collected a wealth of longitudinal phenotype data including information necessary to diagnose COPD and monitor changes in weight and body mass index.

The Jackson Heart Study (JHS)^5^ is an extension of the Atherosclerosis Risk in Communities (ARIC) study with new and continuing enrollment of African-American participants, aged 35–84 years, for observation of cardiovascular risk factors. The study thus included participants from ARIC (31%), from volunteers from counties surrounding Jackson, Mississippi (30%), through random selection (17%), as well as first-degree relatives of index participants (embedded collection of families for genetic study [22%]). Jackson Heart Study did not specifically measure pack-years of cigarette smoking, so smoking history (either “current” or “former”) was used in conjunction with spirometry in the COPD definition for JHS participants.

The Multi-Ethnic Study of Atherosclerosis (MESA)^6^ is a multicenter observational study to identify characteristics of subclinical cardiovascular disease and risk factors for progression to clinical cardiovascular disease. The spirometry used in this study was measured by the MESA Lung Study at Exam 3 or 4 among participants with baseline endothelial function measures, genetic consent and with oversampling of Asians^7^.

The SubPopulations and InteRmediate Outcome Measures In COPD Study (SPIROMICS)^8^ is a longitudinal study which enrolled and followed current and former smokers. Participant data was collected to generate phenotypic, biomarker, genomic and clinical data from subjects with COPD over time.

**SUPPLEMENTAL FIGURE LEGENDS**

**Figure S1. Single Variant Association Testing for Weight Loss in Black/African-American (B/AA) Participants with COPD in the All of Us Research Program.**  A) Analysis design, including analysis method (SAIGE - Scalable and Accurate Implementation of GEneralized mixed model) and case/control counts. B) Quantile-quantile plot of single variant results. C) Manhattan plot of single variant results. Any genome-wide significant results are identified with their position.

**Figure S2. Single Variant Association Testing for Weight Loss in All Participants with COPD in the All of Us Research Program.**  A) Analysis design, including analysis method (SAIGE - Scalable and Accurate Implementation of GEneralized mixed model) and case/control counts. B) Quantile-quantile plot of single variant results. C) Manhattan plot of single variant results. Any genome-wide significant results are identified with their position.

**Figure S3. Single Variant Association Testing for Weight Loss in non-Hispanic white (NHW) Participants with COPD in the All of Us Research Program.**  A) Analysis design, including analysis method (SAIGE - Scalable and Accurate Implementation of GEneralized mixed model) and case/control counts. B) Quantile-quantile plot of single variant results. C) Manhattan plot of single variant results. Any genome-wide significant results are identified with their position.

**Figure S4. Single Variant Association Testing for Weight Loss in All Participants with COPD in the Cardiovascular Health Study (CHS).**  A) Analysis design, including analysis method (GENESIS - GENetic EStimation and Inference in Structured samples) and case/control counts. B) Quantile-quantile plot of single variant results. C) Manhattan plot of single variant results. Any genome-wide significant results are identified with their position.

**Figure S5. Single Variant Association Testing for Weight Loss in Black/African-American (B/AA) Participants with COPD in the Genetic Epidemiology of COPD (COPDGene) study.**  A) Analysis design, including analysis method (GENESIS - GENetic EStimation and Inference in Structured samples) and case/control counts. B) Quantile-quantile plot of single variant results. C) Manhattan plot of single variant results. Any genome-wide significant results are identified with their position.

**Figure S6. Single Variant Association Testing for Weight Loss in non-Hispanic white (NHW) Participants with COPD in the Genetic Epidemiology of COPD (COPDGene) study.**  A) Analysis design, including analysis method (GENESIS - GENetic EStimation and Inference in Structured samples) and case/control counts. B) Quantile-quantile plot of single variant results. C) Manhattan plot of single variant results. Any genome-wide significant results are identified with their position.

**Figure S7. Single Variant Association Testing for Weight Loss in All Participants with COPD in the Evaluation of COPD to Longitudinally Identify Predictive Surrogate Endpoints (ECLIPSE) study.**  A) Analysis design, including analysis method (GENESIS - GENetic EStimation and Inference in Structured samples) and case/control counts. B) Quantile-quantile plot of single variant results. C) Manhattan plot of single variant results. Any genome-wide significant results are identified with their position.

**Figure S8. Single Variant Association Testing for Weight Loss in All Participants with COPD in the Framingham Heart Study (FHS).**  A) Analysis design, including analysis method (GENESIS - GENetic EStimation and Inference in Structured samples) and case/control counts. B) Quantile-quantile plot of single variant results. C) Manhattan plot of single variant results. Any genome-wide significant results are identified with their position.

**Figure S9. Single Variant Association Testing for Weight Loss in All Participants with COPD in the Jackson Heart Study (JHS).**  A) Analysis design, including analysis method (GENESIS - GENetic EStimation and Inference in Structured samples) and case/control counts. B) Quantile-quantile plot of single variant results. C) Manhattan plot of single variant results. Any genome-wide significant results are identified with their position.

**Figure S10. Single Variant Association Testing for Weight Loss in All Participants with COPD in the Multi-Ethnic Study of Atherosclerosis (MESA).** A) Analysis design, including analysis method (GENESIS - GENetic EStimation and Inference in Structured samples) and case/control counts. B) Quantile-quantile plot of single variant results. C) Manhattan plot of single variant results. Any genome-wide significant results are identified with their position.

**Figure S11. Single Variant Association Testing for Weight Loss in Black/African-American (B/AA) Participants with COPD in the SubPopulations and InteRmediate Outcome Measures in COPD Study (SPIROMICS) study.** A) Analysis design, including analysis method (GENESIS - GENetic EStimation and Inference in Structured samples) and case/control counts. B) Quantile-quantile plot of single variant results. C) Manhattan plot of single variant results. Any genome-wide significant results are identified with their position.

**Figure S12. Single Variant Association Testing for Weight Loss in non-Hispanic white (NHW) Participants with COPD in the SubPopulations and InteRmediate Outcome Measures in COPD Study (SPIROMICS) study.** A) Analysis design, including analysis method (GENESIS - GENetic EStimation and Inference in Structured samples) and case/control counts. B) Quantile-quantile plot of single variant results. C) Manhattan plot of single variant results. Any genome-wide significant results are identified with their position.

**Figure S13. Single Variant Association Testing for Weight Loss in All Participants with COPD in the Trans-Omics for Precision Medicine (TOPMed) Initiative.** A) Analysis design, including analysis method (GENESIS - GENetic EStimation and Inference in Structured samples) and case/control counts. B) Quantile-quantile plot of single variant results. C) Manhattan plot of single variant results. Any genome-wide significant results are identified with their position.

**Figure S14. Fine-Mapping Results from Single Variant Analyses of Single Populations.** Credible set variants from PAINTOR (upper panel) and regional associated plots from LocusZoom (lower panel) of the chromosome 5 significant single variant in the *All of Us* Black/African-American cohort (**A**); the chromosome 3 significant single variant in the *All of Us* cosmopolitan cohort (**B**); and the chromosome 3 significant single variant in the *All of Us* non-Hispanic white cohort (**C**). Annotation tracks used in the PAINTOR analysis are listed in the lower portion of the upper panel, while nearby protein-coding genes are shown below the LocusZoom regional association plots.

**Figure S15. Meta-Analysis of Rare Variant Aggregate Association Testing Results from Black/African-American (B/AA) Participants from the Trans-Omics for Precision Medicine (TOPMed) Initiative and *All of Us* Research Program.** A) Analysis design, including analysis method (SAIGE-GENE, followed by Fisher’s method of combining p-values) and case/control counts. B) Quantile-quantile plot of rare variant aggregate testing meta-analysis results. C) Manhattan plot of rare variant aggregate testing meta-analysis results. Any genes meeting the genome-wide significance threshold (p < 2.5x10^-6^) are identified with their gene name.

**Figure S16. Meta-Analysis of Rare Variant Aggregate Association Testing Results All Participants from the Trans-Omics for Precision Medicine (TOPMed) Initiative and *All of Us* Research Program.** A) Analysis design, including analysis method (SAIGE-GENE, followed by Fisher’s method of combining p-values) and case/control counts. B) Quantile-quantile plot of rare variant aggregate testing meta-analysis results. C) Manhattan plot of rare variant aggregate testing meta-analysis results. Any genes meeting the genome-wide significance threshold (p < 2.5x10^-6^) are identified with their gene name.

**Figure S17. Meta-Analysis of Rare Variant Aggregate Association Testing Results from non-Hispanic white (NHW) Participants from the Trans-Omics for Precision Medicine (TOPMed) Initiative and *All of Us* Research Program.** A) Analysis design, including analysis method (SAIGE-GENE, followed by Fisher’s method of combining p-values) and case/control counts. B) Quantile-quantile plot of rare variant aggregate testing meta-analysis results. C) Manhattan plot of rare variant aggregate testing meta-analysis results. Any genes meeting the genome-wide significance threshold (p < 2.5x10^-6^) are identified with their gene name.

**Figure S18. Rare Variant Aggregate Association Testing Results from Black/African-American (B/AA) Participants from the *All of Us* Research Program.** A) Analysis design, including analysis method (SAIGE-GENE) and case/control counts. B) Quantile-quantile plot of rare variant aggregate testing meta-analysis results. C) Manhattan plot of rare variant aggregate testing meta-analysis results. Any genes meeting the genome-wide significance threshold (p < 2.5x10^-6^) are identified with their gene name.

**Figure S19. Rare Variant Aggregate Association Testing Results from All Participants from the *All of Us* Research Program.** A) Analysis design, including analysis method (SAIGE-GENE) and case/control counts. B) Quantile-quantile plot of rare variant aggregate testing meta-analysis results. C) Manhattan plot of rare variant aggregate testing meta-analysis results. Any genes meeting the genome-wide significance threshold (p < 2.5x10^-6^) are identified with their gene name.

**Figure S20. Rare Variant Aggregate Association Testing Results from non-Hispanic white (NHW) Participants from the *All of Us* Research Program.** A) Analysis design, including analysis method (SAIGE-GENE) and case/control counts. B) Quantile-quantile plot of rare variant aggregate testing meta-analysis results. C) Manhattan plot of rare variant aggregate testing meta-analysis results. Any genes meeting the genome-wide significance threshold (p < 2.5x10^-6^) are identified with their gene name.

**Figure S21. Rare Variant Aggregate Association Testing Results from Black/African-American (B/AA) Participants from the Trans-Omics for Precision Medicine (TOPMed) Initiative.** A) Analysis design, including analysis method (SAIGE-GENE) and case/control counts. B) Quantile-quantile plot of rare variant aggregate testing meta-analysis results. C) Manhattan plot of rare variant aggregate testing meta-analysis results. Any genes meeting the genome-wide significance threshold (p < 2.5x10^-6^) are identified with their gene name.

**Figure S22. Rare Variant Aggregate Association Testing Results from All Participants from the Trans-Omics for Precision Medicine (TOPMed) Initiative.** A) Analysis design, including analysis method (SAIGE-GENE) and case/control counts. B) Quantile-quantile plot of rare variant aggregate testing meta-analysis results. C) Manhattan plot of rare variant aggregate testing meta-analysis results. Any genes meeting the genome-wide significance threshold (p < 2.5x10^-6^) are identified with their gene name.

**Figure S23. Rare Variant Aggregate Association Testing Results from non-Hispanic white (NHW) Participants from the Trans-Omics for Precision Medicine (TOPMed) Initiative.** A) Analysis design, including analysis method (SAIGE-GENE) and case/control counts. B) Quantile-quantile plot of rare variant aggregate testing meta-analysis results. C) Manhattan plot of rare variant aggregate testing meta-analysis results. Any genes meeting the genome-wide significance threshold (p < 2.5x10^-6^) are identified with their gene name.

**Figure S24. Predicted Genetically-regulated Gene Expression Effects of Meta-Analyzed Single Variant Associations with Weight Loss in Black/African-American (B/AA) Participants with COPD from MetaXcan.** Genes meeting nominal genome-wide significance (p < 10^-5^) are identified by name.

**Figure S25. Predicted Genetically-regulated Gene Expression Effects of Meta-Analyzed Single Variant Associations with Weight Loss in All (COSMO) Participants with COPD from MetaXcan.** Genes meeting nominal genome-wide significance (p < 10^-5^) are identified by name.

**Figure S26. Predicted Genetically-regulated Gene Expression Effects of Meta-Analyzed Single Variant Associations with Weight Loss in non-Hispanic white (NHW) Participants with COPD from MetaXcan.** Genes meeting nominal genome-wide significance (p < 10^-5^) are identified by name.

**Figure S27. Regional Association Plots of Single Variant Associations with Weight Loss in COPD near Previously-Implicated Genes**. Gene names are found along the bottom panel with exons represented by filled-in boxes. P-values are plotted on a negative log scale on the y axis and each dot represents a variant.

**SUPPLEMENTAL REFERENCES**

1. Fried, L.P. *et al.* The Cardiovascular Health Study: design and rationale. *Ann Epidemiol* **1**, 263-76 (1991).

2. Regan, E.A. *et al.* Genetic Epidemiology of COPD (COPDGene) Study Design. *COPD: Journal of Chronic Obstructive Pulmonary Disease* **7**, 32-43 (2011).

3. Vestbo, J. *et al.* Evaluation of COPD Longitudinally to Identify Predictive Surrogate End-points (ECLIPSE). *European Respiratory Journal* **31**, 869-873 (2008).

4. Splansky, G.L. *et al.* The Third Generation Cohort of the National Heart, Lung, and Blood Institute's Framingham Heart Study: Design, Recruitment, and Initial Examination. *American Journal of Epidemiology* **165**, 1328-1335 (2007).

5. Sempos, C.T., Bild, D.E. & Manolio, T.A. Overview of the Jackson Heart Study: a study of cardiovascular diseases in African American men and women. *Am J Med Sci* **317**, 142-6 (1999).

6. Bild, D.E. Multi-Ethnic Study of Atherosclerosis: Objectives and Design. *American Journal of Epidemiology* **156**, 871-881 (2002).

7. Rodriguez, J. *et al.* The Association of Pipe and Cigar Use With Cotinine Levels, Lung Function, and Airflow Obstruction. *Annals of Internal Medicine* **152**, 201-210 (2010).

8. Couper, D. *et al.* Design of the Subpopulations and Intermediate Outcomes in COPD Study (SPIROMICS): Table 1. *Thorax* **69**, 492-495 (2014).

**SUPPLEMENTAL ACKNOWLEDGEMENTS**

**TOPMed: Cardiovascular Health Study** This research was supported by contracts HHSN268201200036C, HHSN268200800007C, HHSN268201800001C, N01-HC85079, N01-HC-85080, N01-HC-85081, N01-HC-85082, N01-HC-85083, N01-HC-85084, N01-HC-85085, N01-HC-85086, N01-HC-35129, N01-HC-15103, N01-HC-55222, N01-HC-75150, N01-HC-45133, and N01-HC-85239; grant numbers U01 HL080295, U01 HL130114 and R01 HL059367 from the National Heart, Lung, and Blood Institute, and R01 AG023629 from the National Institute on Aging, with additional contributions from the National Institute of Neurological Disorders and Stroke. A full list of principal CHS investigators and institutions can be found at https://chs-nhlbi.org/pi. Its content is solely the responsibility of the authors and does not necessarily represent the official views of the National Institutes of Health.

**TOPMed: Genetic Epidemiology of COPD (COPDGene) in the TOPMed Program** This research used data generated by the COPDGene study, which was supported by NIH Award Number U01 HL089897 and Award Number U01 HL089856 from the National Heart, Lung, and Blood Institute. The content is solely the responsibility of the authors and does not necessarily represent the official views of the National Heart, Lung, and Blood Institute or the National Institutes of Health. The COPDGene project is also supported by the COPD Foundation through contributions made to an Industry Advisory Board comprised of AstraZeneca, Boehringer Ingelheim, GlaxoSmithKline, Novartis, Pfizer, Siemens and Sunovion.

**TOPMed: The ECLIPSE Study** The ECLIPSE study (NCT00292552) was sponsored by GlaxoSmithKline.
The ECLIPSE investigators included: Bulgaria: Y. Ivanov, Pleven; K. Kostov, Sofia. Canada: J. Bourbeau, Montreal; M. Fitzgerald, Vancouver, BC; P. Hernandez, Halifax, NS; K. Killian, Hamilton, ON; R. Levy, Vancouver, BC; F. Maltais, Montreal; D. O'Donnell, Kingston, ON. Czech Republic: J. Krepelka, Prague. Denmark: J. Vestbo, Hvidovre. The Netherlands: E. Wouters, Horn-Maastricht. New Zealand: D. Quinn, Wellington. Norway: P. Bakke, Bergen. Slovenia: M. Kosnik, Golnik. Spain: A. Agusti, J. Sauleda, P. de Mallorca. Ukraine: Y. Feschenko, V. Gavrisyuk, L. Yashina, Kiev; N. Monogarova, Donetsk. United Kingdom: P. Calverley, Liverpool; D. Lomas, Cambridge; W. MacNee, Edinburgh; D. Singh, Manchester; J. Wedzicha, London. United States: A. Anzueto, San Antonio, TX; S. Braman, Providence, RI; R. Casaburi, Torrance CA; B. Celli, Boston; G. Giessel, Richmond, VA; M. Gotfried, Phoenix, AZ; G. Greenwald, Rancho Mirage, CA; N. Hanania, Houston; D. Mahler, Lebanon, NH; B. Make, Denver; S. Rennard, Omaha, NE; C. Rochester, New Haven, CT; P. Scanlon, Rochester, MN; D. Schuller, Omaha, NE; F. Sciurba, Pittsburgh; A. Sharafkhaneh, Houston; T. Siler, St. Charles, MO; E. Silverman, Boston; A. Wanner, Miami; R. Wise, Baltimore; R. ZuWallack, Hartford, CT.
ECLIPSE Steering Committee: H. Coxson (Canada), C. Crim (GlaxoSmithKline, USA), L. Edwards (GlaxoSmithKline, USA), D. Lomas (UK), W. MacNee (UK), E. Silverman (USA), R. Tal-Singer (Co-chair, GlaxoSmithKline, USA), J. Vestbo (Co-chair, Denmark), J. Yates (GlaxoSmithKline, USA).
ECLIPSE Scientific Committee: A. Agusti (Spain), P. Calverley (UK), B. Celli (USA), C. Crim (GlaxoSmithKline, USA), B. Miller (GlaxoSmithKline, USA), W. MacNee (Chair, UK), S. Rennard (USA), R. Tal-Singer (GlaxoSmithKline, USA), E. Wouters (The Netherlands), J. Yates (GlaxoSmithKline, USA).

**TOPMed: Whole Genome Sequencing and Related Phenotypes in the Framingham Heart Study** The Framingham Heart Study (FHS) acknowledges the support of contracts NO1-HC-25195 and HHSN268201500001I from the National Heart, Lung, and Blood Institute and grant supplement R01 HL092577-06S1 for this research. We also acknowledge the dedication of the FHS study participants without whom this research would not be possible. Dr. Vasan is supported in part by the Evans Medical Foundation and the Jay and Louis Coffman Endowment from the Department of Medicine, Boston University School of Medicine.

**TOPMed: The Jackson Heart Study** The Jackson Heart Study (JHS) is supported and conducted in collaboration with Jackson State University (HHSN268201800013I), Tougaloo College (HHSN268201800014I), the Mississippi State Department of Health (HHSN268201800015I/HHSN26800001) and the University of Mississippi Medical Center (HHSN268201800010I, HHSN268201800011I and HHSN268201800012I) contracts from the National Heart, Lung, and Blood Institute (NHLBI) and the National Institute for Minority Health and Health Disparities (NIMHD). The authors also wish to thank the staff and participants of the JHS.

**TOPMed: Multi-Ethnic Study of Atherosclerosis** MESA and the MESA SHARe projects are conducted and supported by the National Heart, Lung, and Blood Institute (NHLBI) in collaboration with MESA investigators. Support for MESA is provided by contracts 75N92020D00001, HHSN268201500003I, N01-HC-95159, 75N92020D00005, N01-HC-95160, 75N92020D00002, N01-HC-95161, 75N92020D00003, N01-HC-95162, 75N92020D00006, N01-HC-95163, 75N92020D00004, N01-HC-95164, 75N92020D00007, N01-HC-95165, N01-HC-95166, N01-HC-95167, N01-HC-95168, N01-HC-95169, UL1-TR-000040, UL1-TR-001079, UL1-TR-001420. Also supported in part by the National Center for Advancing Translational Sciences, CTSI grant UL1TR001881, and the National Institute of Diabetes and Digestive and Kidney Disease Diabetes Research Center (DRC) grant DK063491 to the Southern California Diabetes Endocrinology Research Center. The MESA Lung Study is funded by R01-HL077612.

**TOPMed:** **SubPopulations and InteRmediate Outcome Measures In COPD Study (SPIROMICS)** The authors thank the SPIROMICS participants and participating physicians, investigators and staff for making this research possible. More information about the study and how to access SPIROMICS data is at www.spiromics.org. We would like to acknowledge the following current and former investigators of the SPIROMICS sites and reading centers: Neil E Alexis, MD; Wayne H Anderson, PhD; Mehrdad Arjomandi, MD; Igor Barjaktarevic, MD, PhD; R Graham Barr, MD, DrPH; Lori A Bateman, MSc; Surya P Bhatt, MD; Eugene R Bleecker, MD; Richard C Boucher, MD; Russell P Bowler, MD, PhD;; Stephanie A Christenson, MD; Alejandro P Comellas, MD; Christopher B Cooper, MD, PhD; David J Couper, PhD; Gerard J Criner, MD; Ronald G Crystal, MD; Jeffrey L Curtis, MD; Claire M Doerschuk, MD; Mark T Dransfield, MD; Brad Drummond, MD; Christine M Freeman, PhD; Craig Galban, PhD; MeiLan K Han, MD, MS; Nadia N Hansel, MD, MPH; Annette T Hastie, PhD; Eric A Hoffman, PhD; Yvonne Huang, MD; Robert J Kaner, MD; Richard E Kanner, MD; Eric C Kleerup, MD; Jerry A Krishnan, MD, PhD; Lisa M LaVange, PhD; Stephen C Lazarus, MD; Fernando J Martinez, MD, MS; Deborah A Meyers, PhD; Wendy C Moore, MD; John D Newell Jr, MD; Robert Paine, III, MD; Laura Paulin, MD, MHS; Stephen P Peters, MD, PhD; Cheryl Pirozzi, MD; Nirupama Putcha, MD, MHS; Elizabeth C Oelsner, MD, MPH; Wanda K O'Neal, PhD; Victor E Ortega, MD, PhD;; Sanjeev Raman, MBBS, MD; Stephen I. Rennard, MD; Donald P Tashkin, MD;; J Michael Wells, MD; Robert A Wise, MD; and Prescott G Woodruff, MD, MPH. The project officers from the Lung Division of the National Heart, Lung, and Blood Institute were Lisa Postow, PhD, and Lisa Viviano, BSN; SPIROMICS was supported by contracts from the NIH/NHLBI (HHSN268200900013C, HHSN268200900014C, HHSN268200900015C, HHSN268200900016C, HHSN268200900017C, HHSN268200900018C, HHSN268200900019C, HHSN268200900020C), and a grant from the NIH/NHLBI (U01 HL137880, and supplemented by contributions made through the Foundation for the NIH and the COPD Foundation from AstraZeneca/MedImmune; Bayer; Bellerophon Therapeutics; BoehringerIngelheim Pharmaceuticals, Inc..; Chiesi Farmaceutici S.p.A.; Forest Research Institute, Inc.; GlaxoSmithKline; Grifols Therapeutics, Inc.; Ikaria, Inc.; Novartis Pharmaceuticals Corporation; Nycomed GmbH; ProterixBio; ; Regeneron Pharmaceuticals, Inc.; Sanofi; Sunovion; Takeda Pharmaceutical Company; and Theravance Biopharma and Mylan.
