# Supplementary figures and images for: Whole Genome Sequence Analysis of Weight Loss in 16 972 Participants With COPD Reveals Novel Risk Loci in DRAIC and RFX3
Source: J Cachexia Sarcopenia Muscle. 2026 Apr 23;17(3):e70293. doi: 10.1002/jcsm.70293 (PMC13106028; doi:10.1002/jcsm.70293)

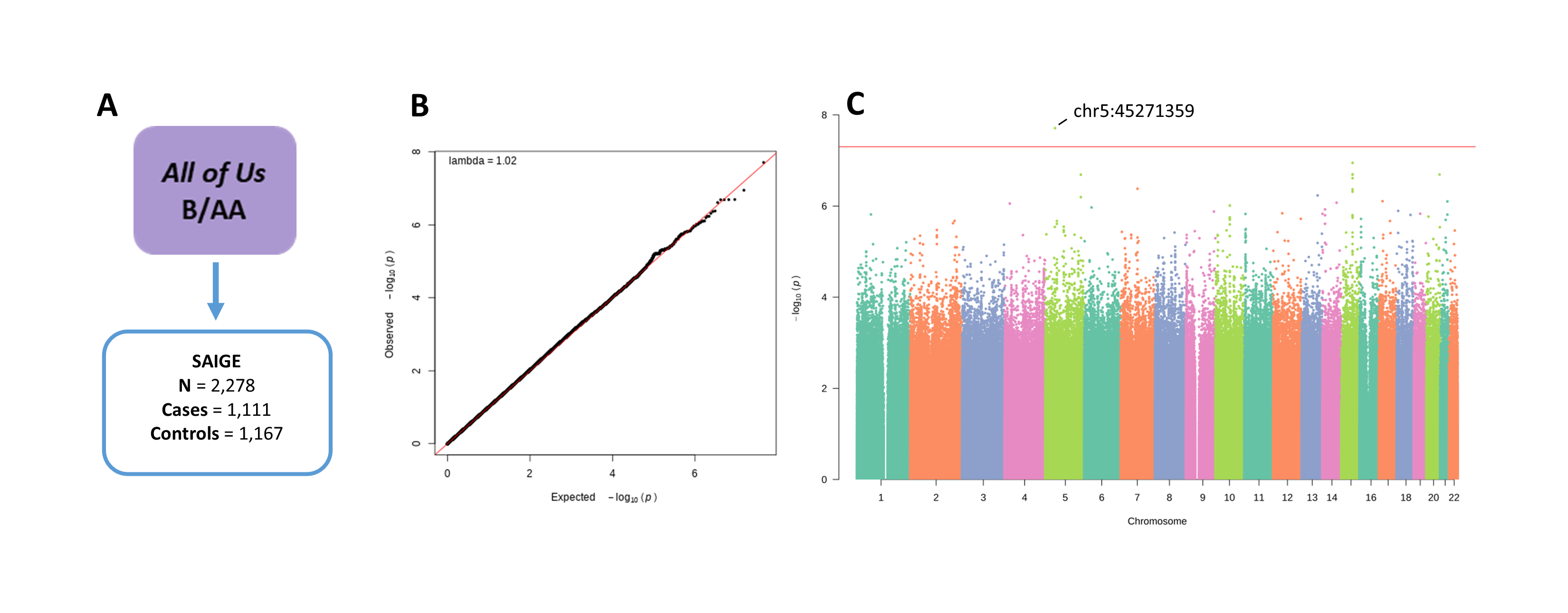

Supplement: Supplementary file 3 — Figure S1: Single variant association testing for weight loss in Black/African–American (B/AA) participants with COPD in the All of Us Research Program. (A) Analysis design, including analysis method (SAIGE, Scalable and Accurate Implementation of GEneralized mixed model) and case/control counts. (B) Quantile–quantile plot of single variant results. (C) Manhattan plot of single variant results. Any genome‐wide significant results are identified with their position. [file JCSM-17-e70293-s022.tif]

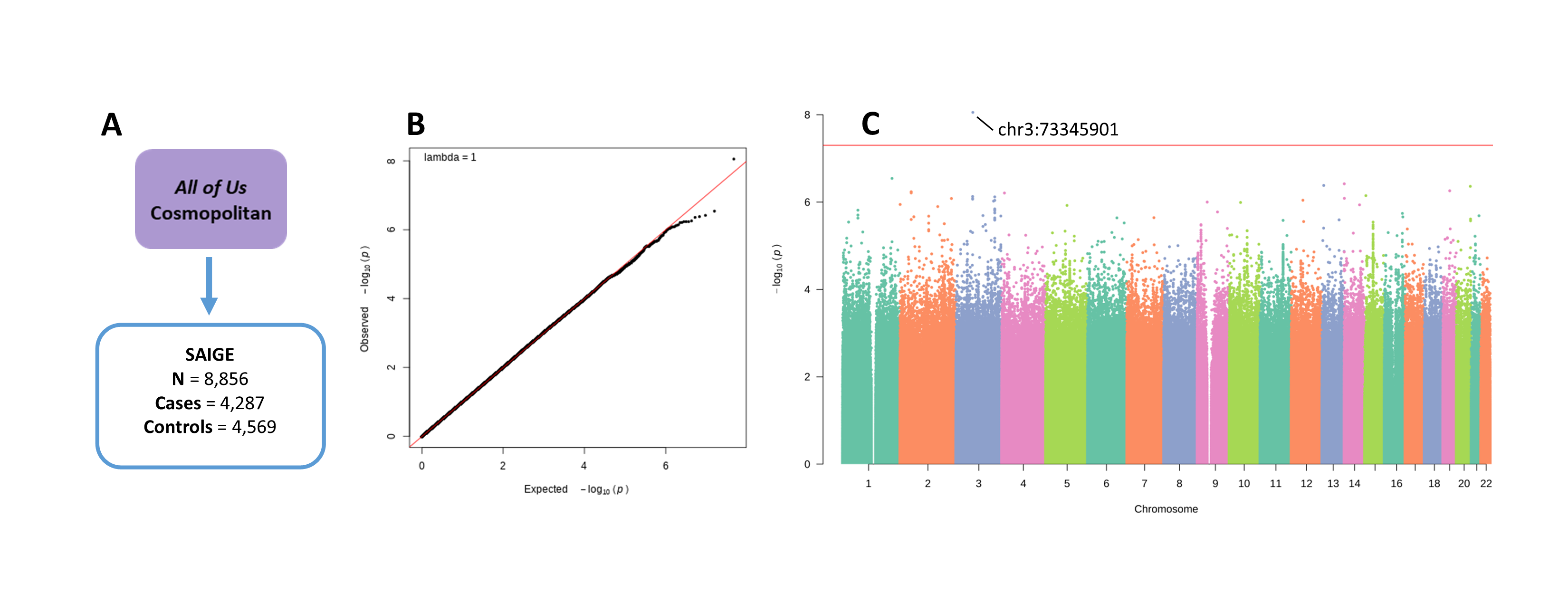

Supplement: Supplementary file 4 — Figure S2: Single Variant Association Testing for Weight Loss in All Participants with COPD in the All of Us Research Program. (A) Analysis design, including analysis method (SAIGE—Scalable and Accurate Implementation of GEneralized mixed model) and case/control counts. (B) Quantile–quantile plot of single variant results. (C) Manhattan plot of single variant results. Any genome‐wide significant results are identified with their position. [file JCSM-17-e70293-s007.tif]

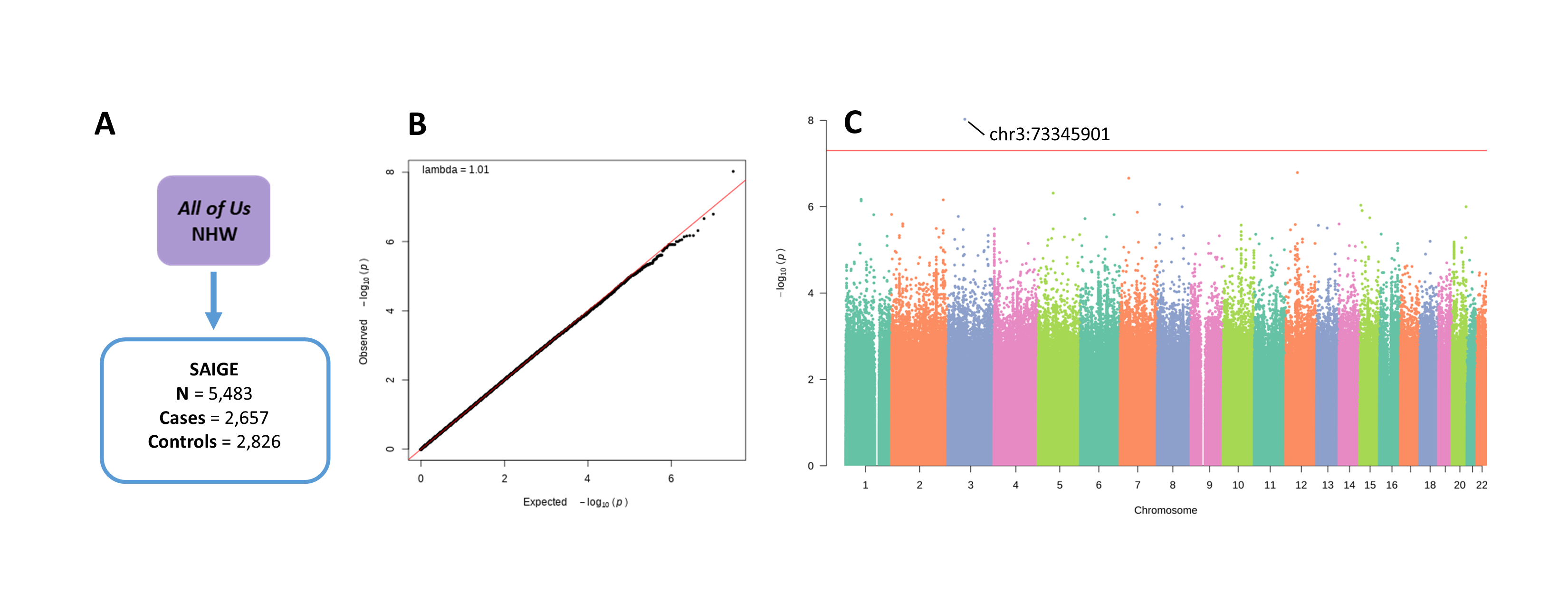

Supplement: Supplementary file 5 — Figure S3: Single Variant Association Testing for Weight Loss in non‐Hispanic white (NHW) Participants with COPD in the All of Us Research Program. (A) Analysis design, including analysis method (SAIGE—Scalable and Accurate Implementation of GEneralized mixed model) and case/control counts. (B) Quantile–quantile plot of single variant results. (C) Manhattan plot of single variant results. Any genome‐wide significant results are identified with their position. [file JCSM-17-e70293-s015.tif]

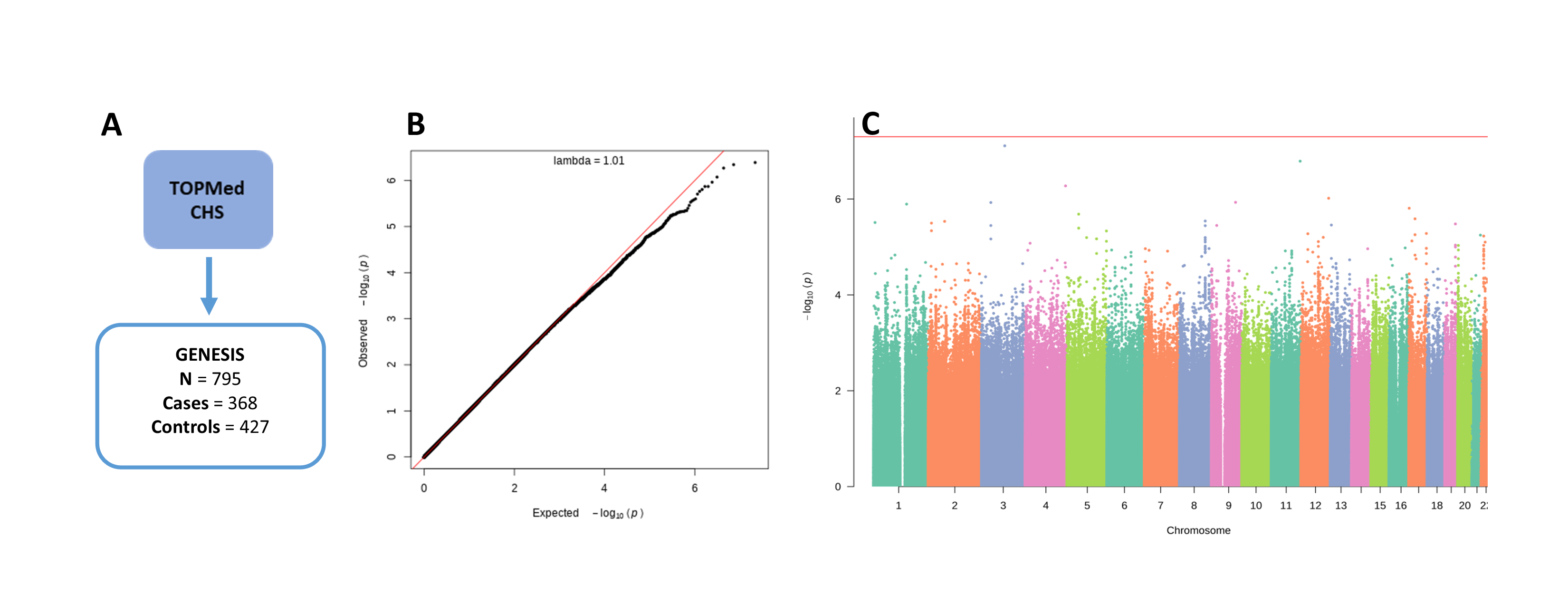

Supplement: Supplementary file 6 — Figure S4: Single variant association testing for weight loss in all participants with COPD in the Cardiovascular Health Study (CHS). (A) Analysis design, including analysis method (GENESIS—GENetic EStimation and Inference in Structured samples) and case/control counts. (B) Quantile–quantile plot of single variant results. (C) Manhattan plot of single variant results. Any genome‐wide significant results are identified with their position. [file JCSM-17-e70293-s014.tif]

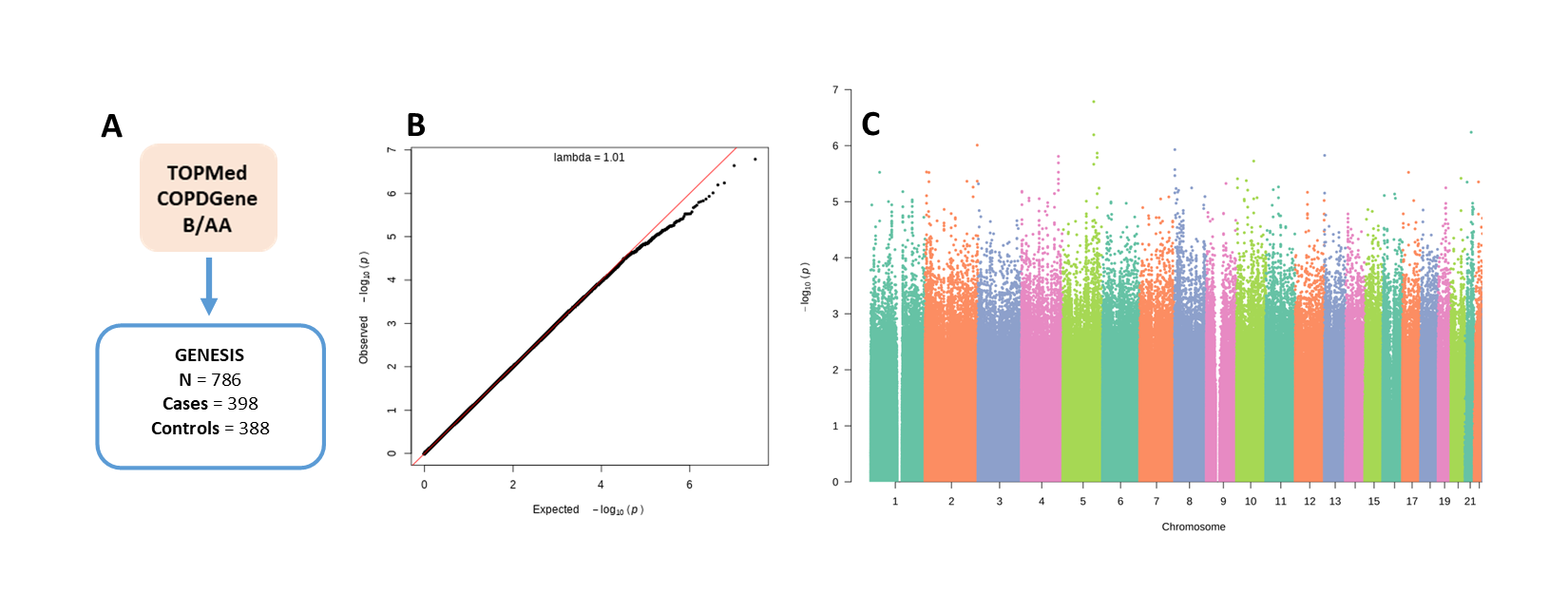

Supplement: Supplementary file 7 — Figure S5: Single variant association testing for weight loss in Black/African–American (B/AA) participants with COPD in the genetic epidemiology of COPD (COPDGene) study. (A) Analysis design, including analysis method (GENESIS—GENetic EStimation and Inference in Structured samples) and case/control counts. (B) Quantile–quantile plot of single variant results. (C) Manhattan plot of single variant results. Any genome‐wide significant results are identified with their position. [file JCSM-17-e70293-s006.tif]

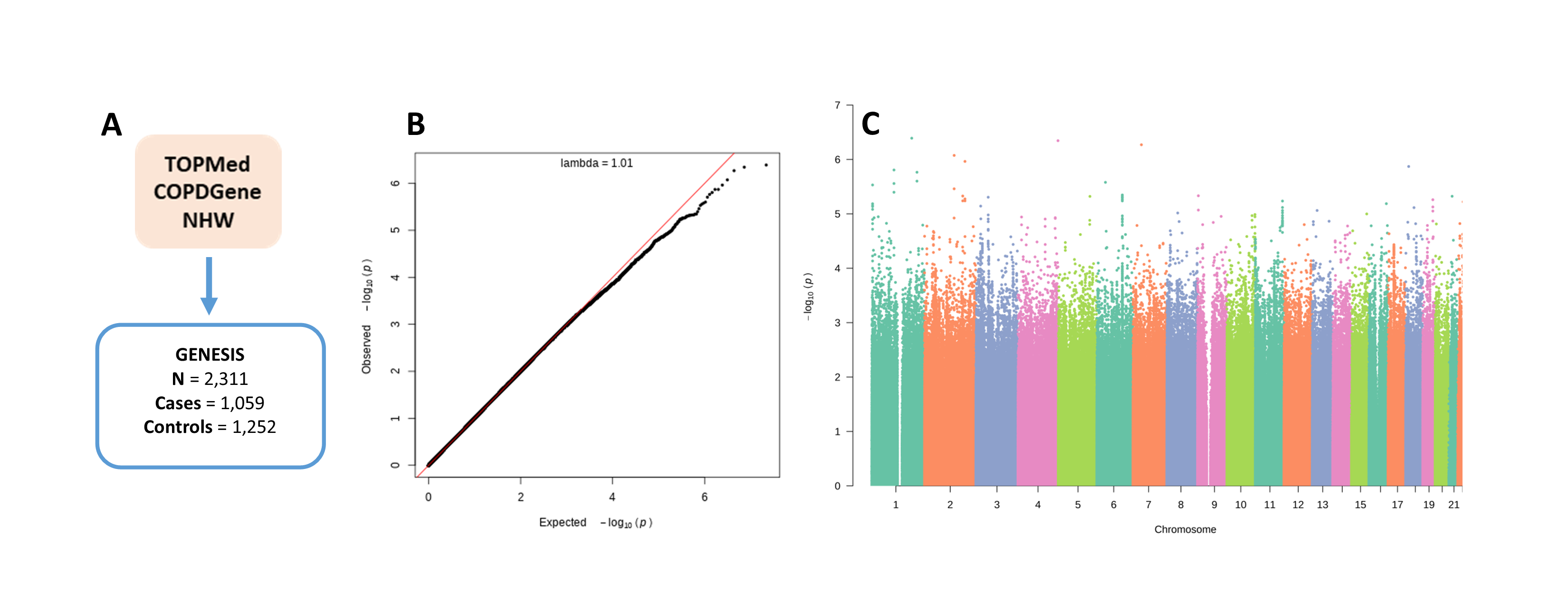

Supplement: Supplementary file 8 — Figure S6: Single variant association testing for weight loss in non‐Hispanic white (NHW) participants with COPD in the genetic epidemiology of COPD (COPDGene) study. (A) Analysis design, including analysis method (GENESIS—GENetic EStimation and Inference in Structured samples) and case/control counts. (B) Quantile–quantile plot of single variant results. (C) Manhattan plot of single variant results. Any genome‐wide significant results are identified with their position. [file JCSM-17-e70293-s016.tif]

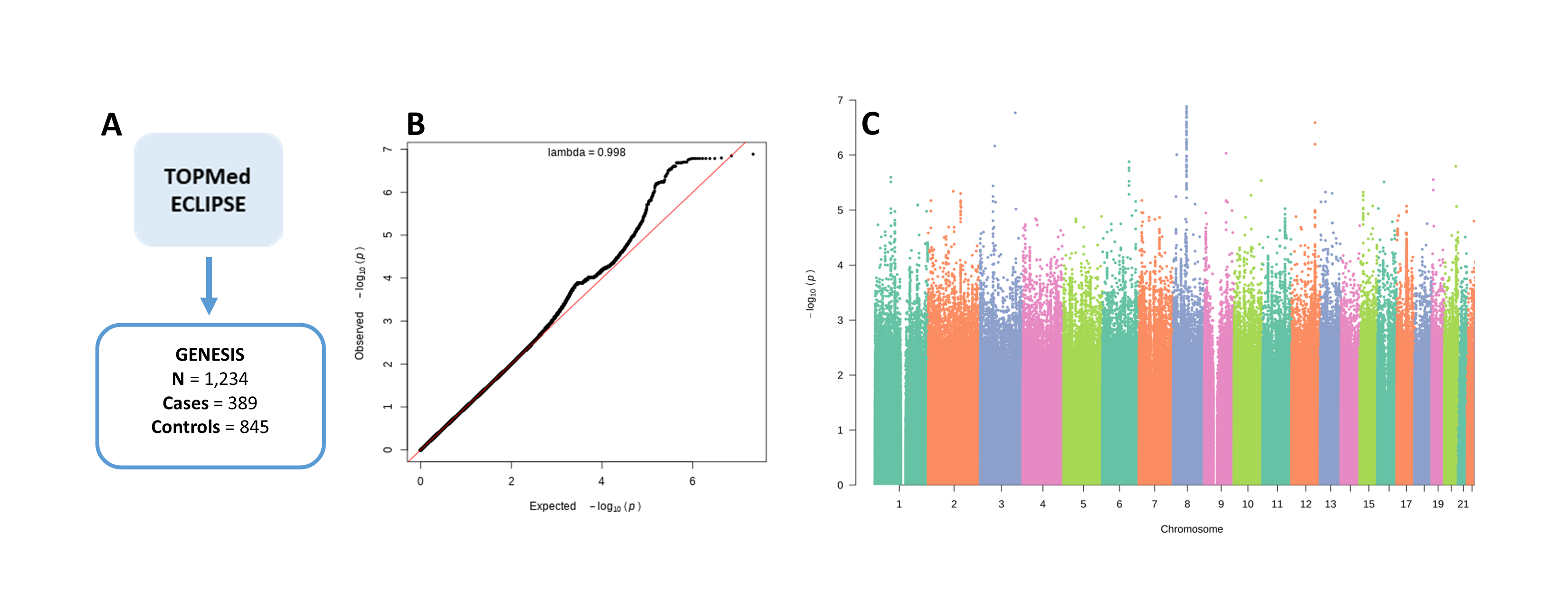

Supplement: Supplementary file 9 — Figure S7: Single variant association testing for weight loss in all participants with COPD in the evaluation of COPD to longitudinally identify predictive surrogate endpoints (ECLIPSE) study. (A) Analysis design, including analysis method (GENESIS—GENetic EStimation and Inference in Structured samples) and case/control counts. (B) Quantile–quantile plot of single variant results. (C) Manhattan plot of single variant results. Any genome‐wide significant results are identified with their position. [file JCSM-17-e70293-s004.tif]

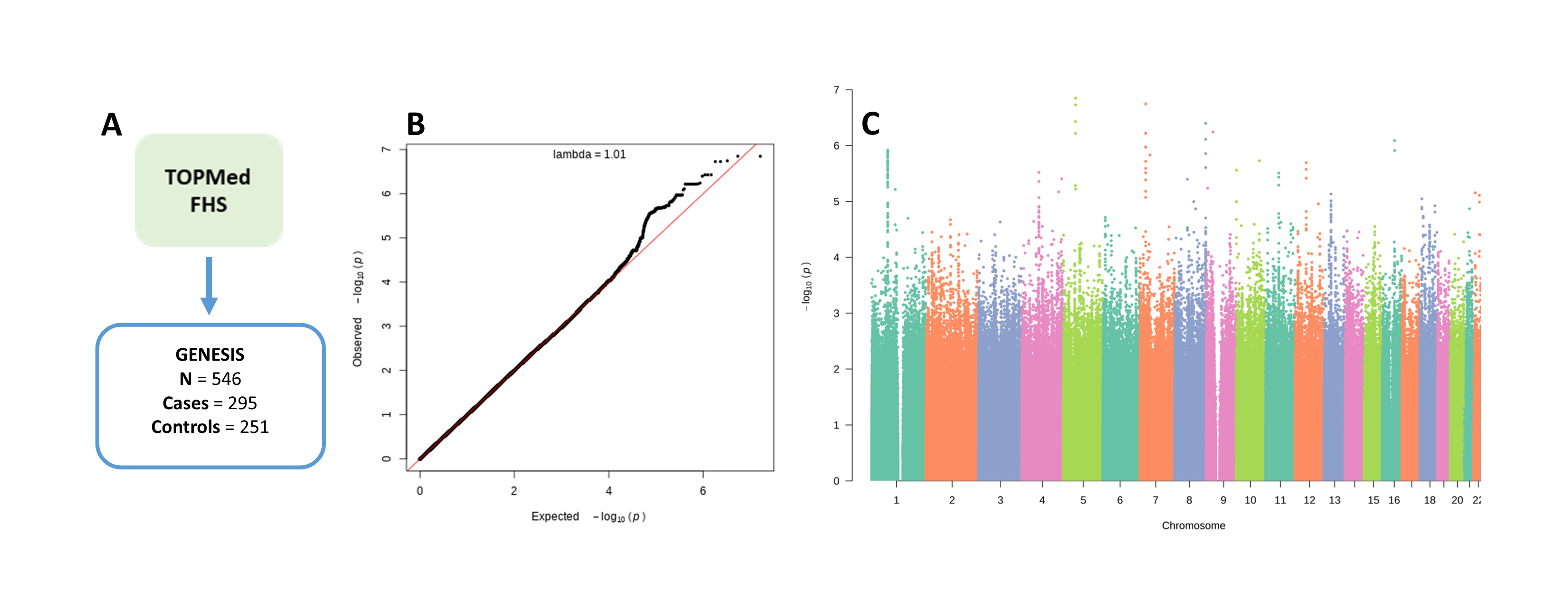

Supplement: Supplementary file 10 — Figure S8: Single variant association testing for weight loss in all participants with COPD in the Framingham Heart Study (FHS). (A) Analysis design, including analysis method (GENESIS—GENetic EStimation and Inference in Structured samples) and case/control counts. (B) Quantile–quantile plot of single variant results. (C) Manhattan plot of single variant results. Any genome‐wide significant results are identified with their position. [file JCSM-17-e70293-s009.tif]

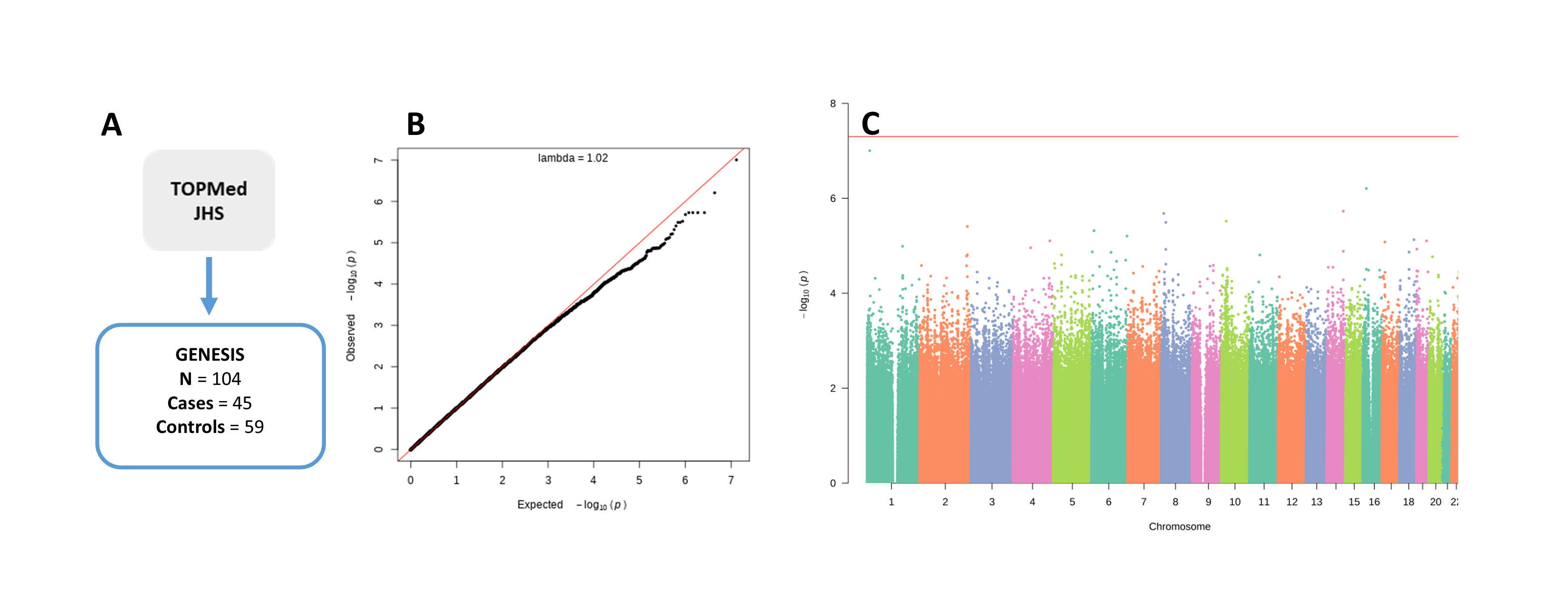

Supplement: Supplementary file 11 — Figure S9: Single variant association testing for weight loss in all participants with COPD in the Jackson Heart Study (JHS). (A) Analysis design, including analysis method (GENESIS—GENetic EStimation and Inference in Structured samples) and case/control counts. (B) Quantile–quantile plot of single variant results. (C) Manhattan plot of single variant results. Any genome‐wide significant results are identified with their position. [file JCSM-17-e70293-s011.tif]

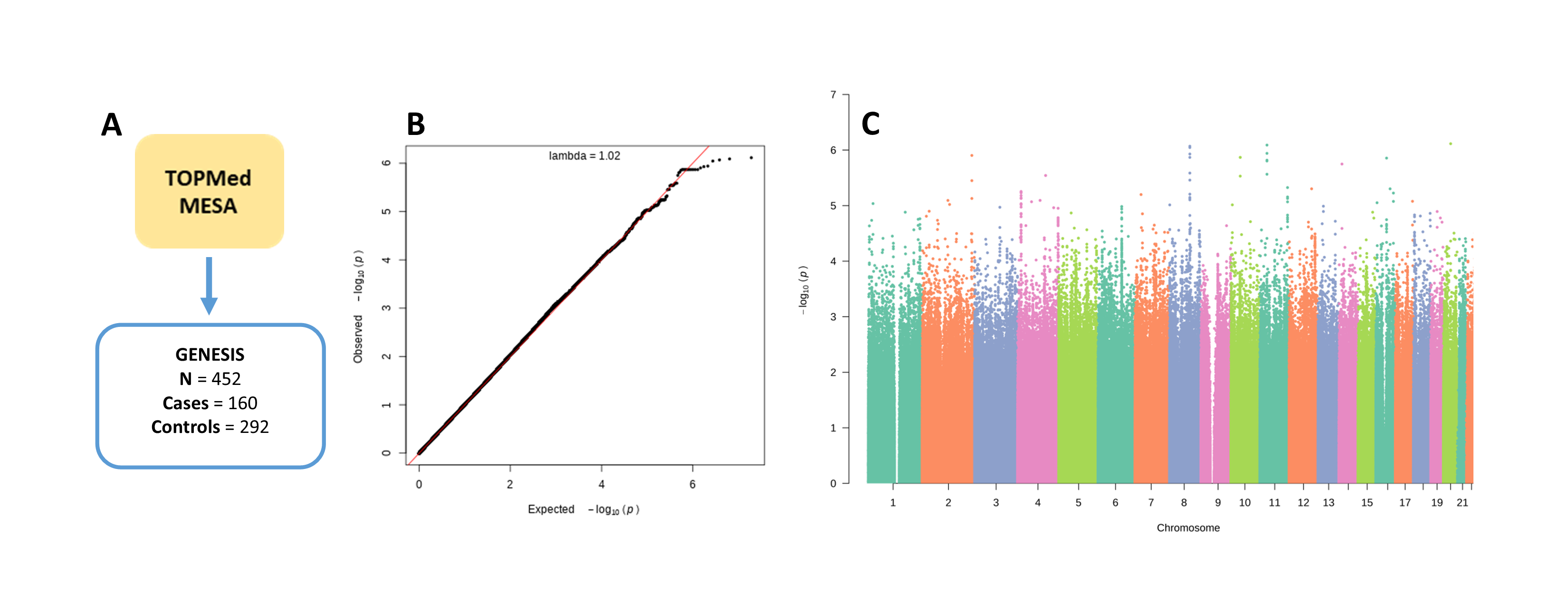

Supplement: Supplementary file 12 — Figure S10: Single variant association testing for weight loss in all participants with COPD in the multi‐ethnic study of atherosclerosis (MESA). (A) Analysis design, including analysis method (GENESIS—GENetic EStimation and Inference in Structured samples) and case/control counts. (B) Quantile–quantile plot of single variant results. (C) Manhattan plot of single variant results. Any genome‐wide significant results are identified with their position. [file JCSM-17-e70293-s017.tif]

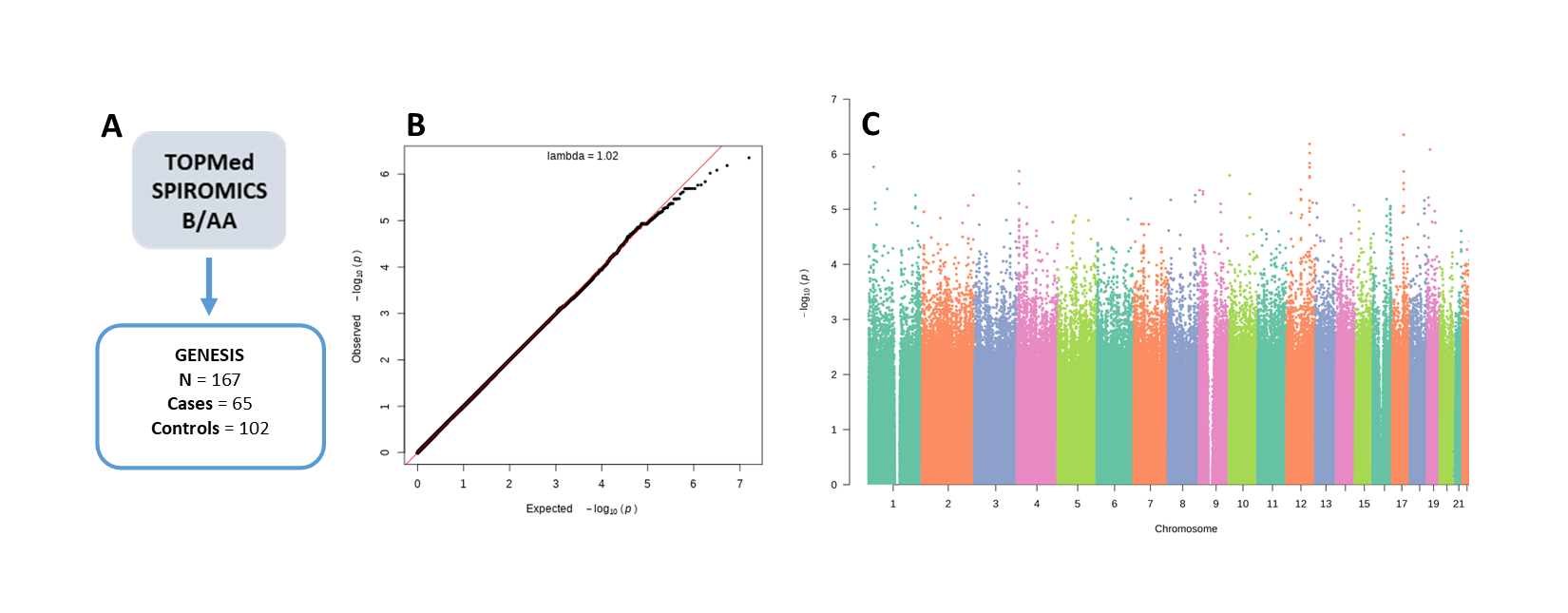

Supplement: Supplementary file 13 — Figure S11: Single variant association testing for weight loss in Black/African–American (B/AA) participants with COPD in the SubPopulations and InteRmediate Outcome Measures in COPD Study (SPIROMICS) study. (A) Analysis design, including analysis method (GENESIS—GENetic EStimation and Inference in Structured samples) and case/control counts. (B) Quantile–quantile plot of single variant results. (C) Manhattan plot of single variant results. Any genome‐wide significant results are identified with their position. [file JCSM-17-e70293-s001.tif]

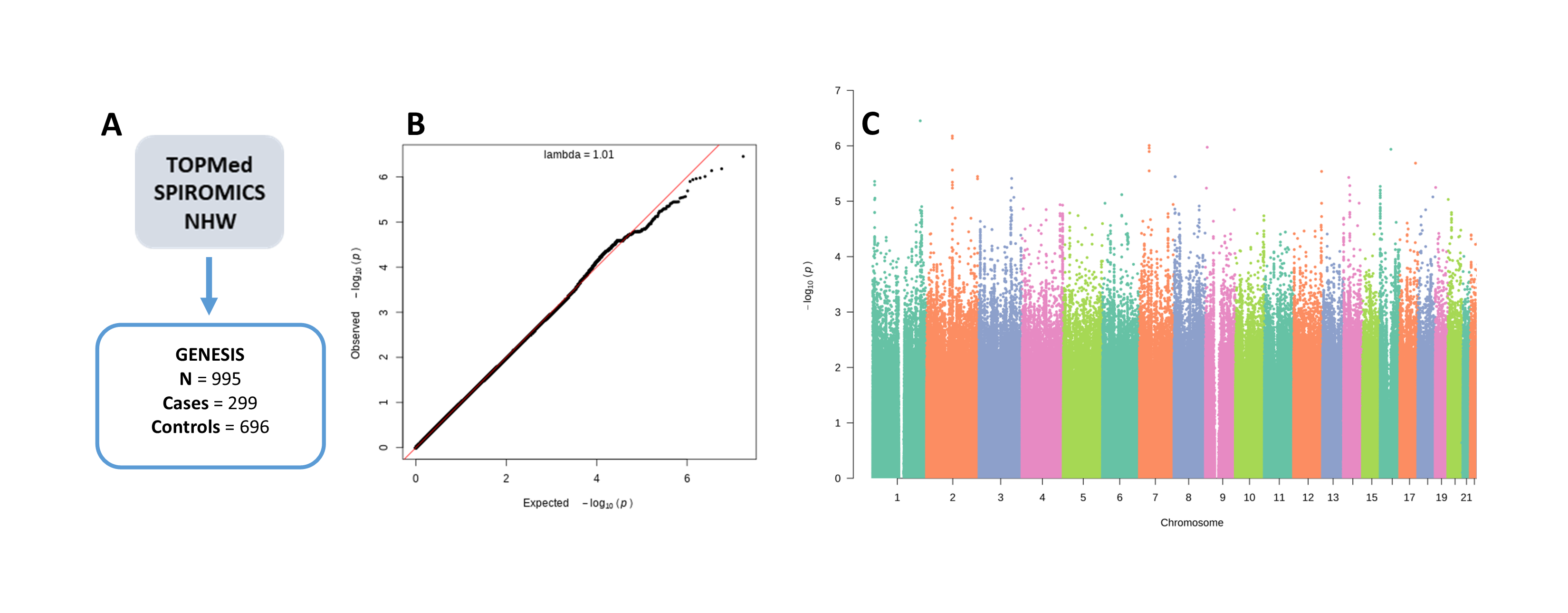

Supplement: Supplementary file 14 — Figure S12: Single variant association testing for weight loss in non‐Hispanic white (NHW) participants with COPD in the SubPopulations and InteRmediate Outcome Measures in COPD Study (SPIROMICS) study. (A) Analysis design, including analysis method (GENESIS—GENetic EStimation and Inference in Structured samples) and case/control counts. (B) Quantile–quantile plot of single variant results. (C) Manhattan plot of single variant results. Any genome‐wide significant results are identified with their position. [file JCSM-17-e70293-s010.tif]

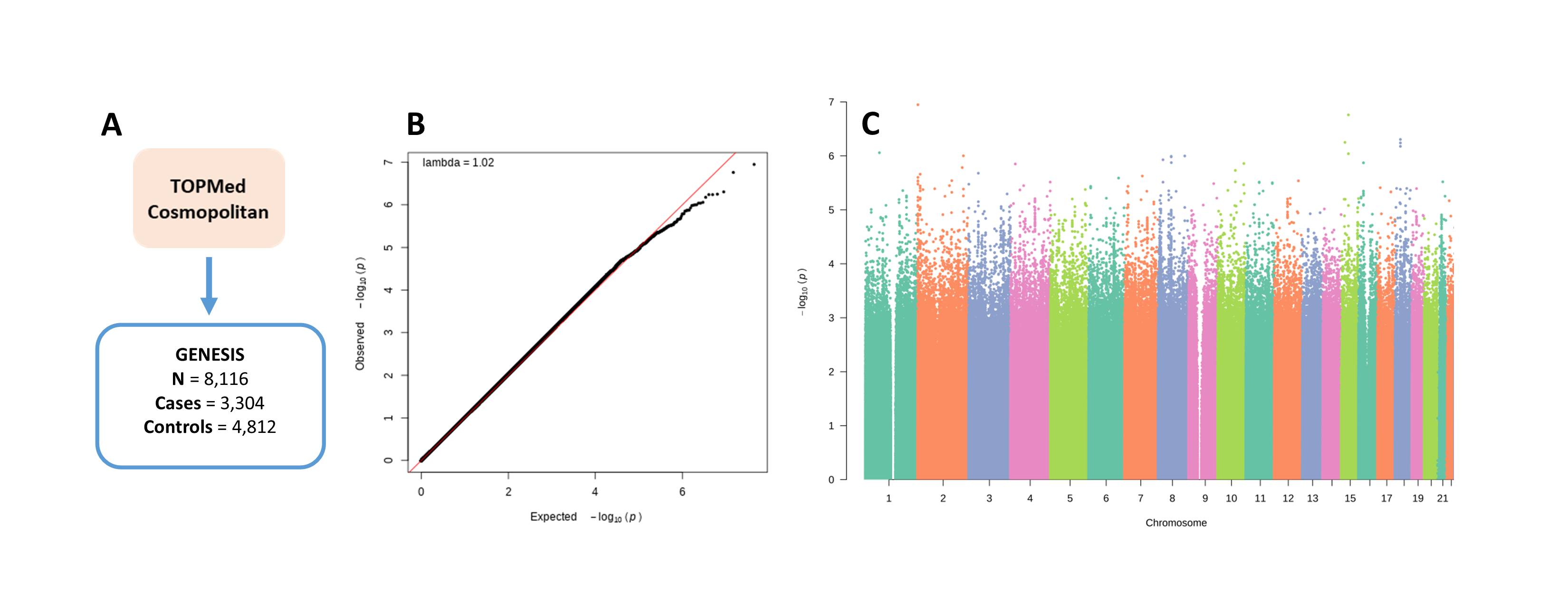

Supplement: Supplementary file 15 — Figure S13: Single variant association testing for weight loss in all participants with COPD in the Trans‐Omics for Precision Medicine (TOPMed) Initiative. (A) Analysis design, including analysis method (GENESIS—GENetic EStimation and Inference in Structured samples) and case/control counts. (B) Quantile–quantile plot of single variant results. (C) Manhattan plot of single variant results. Any genome‐wide significant results are identified with their position. [file JCSM-17-e70293-s002.tif]

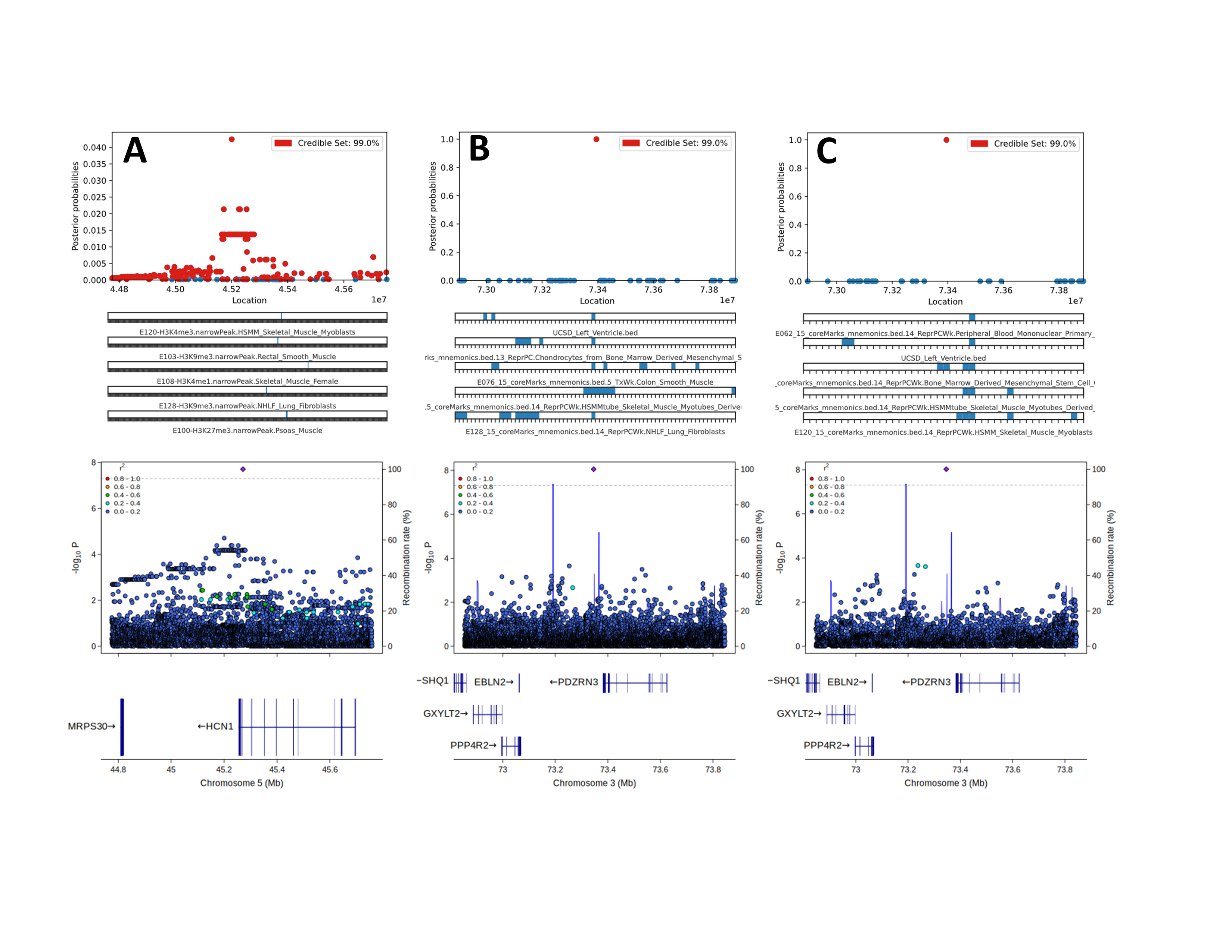

Supplement: Supplementary file 16 — Figure S14: Fine‐mapping results from single variant analyses of single populations. Credible set variants from PAINTOR (upper panel) and regional associated plots from LocusZoom (lower panel) of the chromosome 5 significant single variant in the All of Us Black/African–American cohort (A); the chromosome 3 significant single variant in the All of Us cosmopolitan cohort (B); and the chromosome 3 significant single variant in the All of Us non‐Hispanic white cohort (C). Annotation tracks used in the PAINTOR analysis are listed in the lower portion of the upper panel, while nearby protein‐coding genes are shown below the LocusZoom regional association plots. [file JCSM-17-e70293-s018.tif]

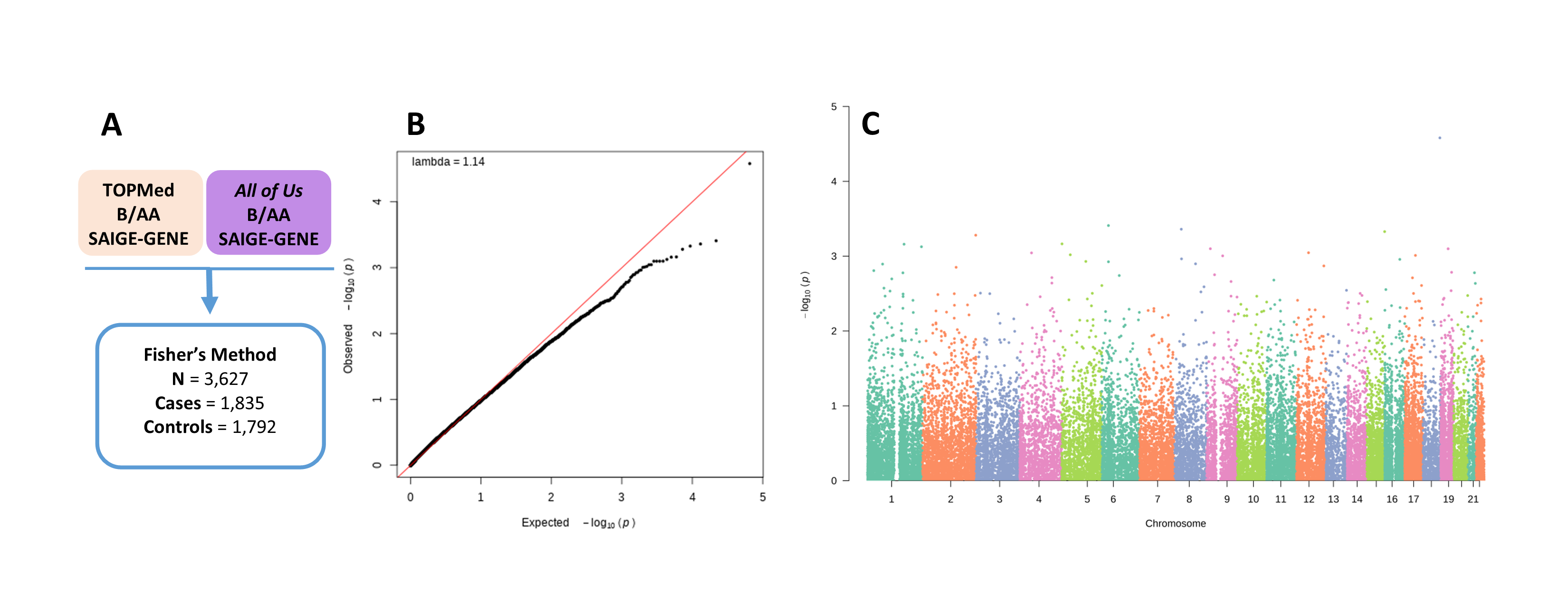

Supplement: Supplementary file 17 — Figure S15: Meta‐analysis of rare variant aggregate association testing results from Black/African–American (B/AA) participants from the Trans‐Omics for Precision Medicine (TOPMed) Initiative and All of Us Research Program. (A) Analysis design, including analysis method (SAIGE‐GENE, followed by Fisher's method of combining p‐values) and case/control counts. (B) Quantile–quantile plot of rare variant aggregate testing meta‐analysis results. (C) Manhattan plot of rare variant aggregate testing meta‐analysis results. Any genes meeting the genome‐wide significance threshold (p < 2.5 × 10^−6^) are identified with their gene name. [file JCSM-17-e70293-s023.tif]

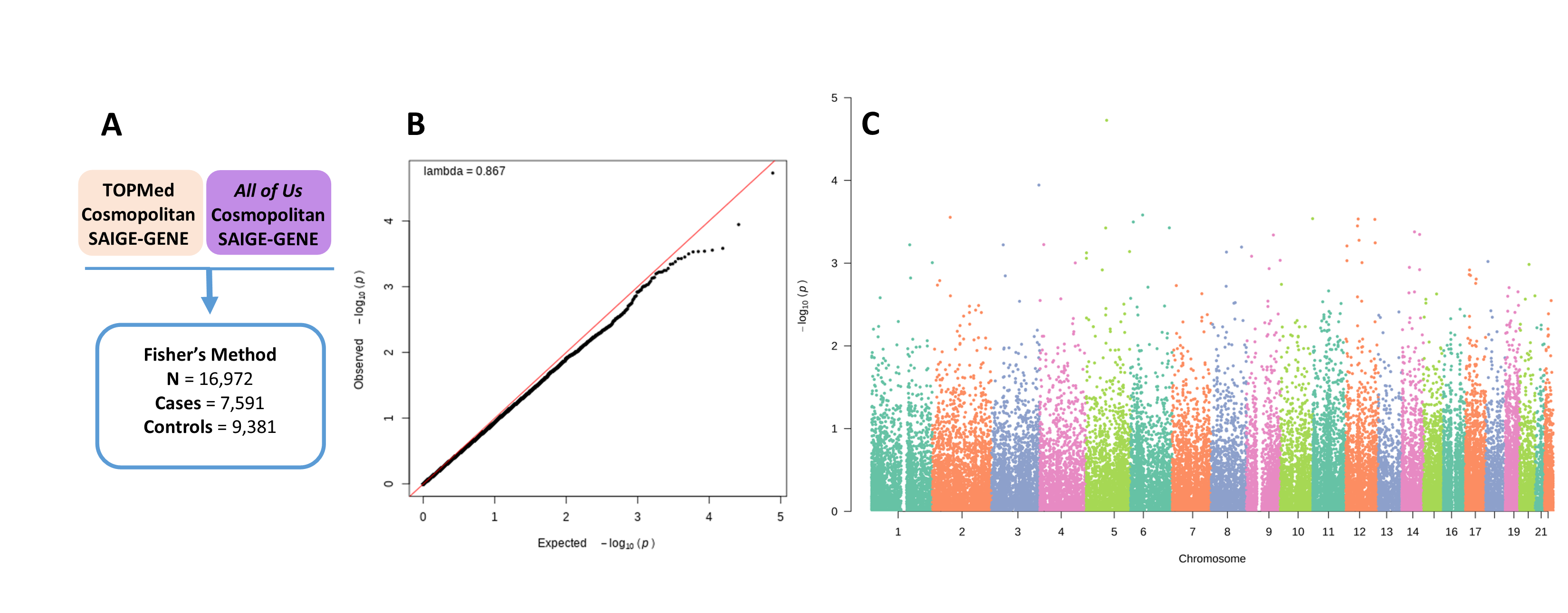

Supplement: Supplementary file 18 — Figure S16: Meta‐analysis of rare variant aggregate association testing results all participants from the Trans‐Omics for Precision Medicine (TOPMed) initiative and All of Us Research Program. (A) Analysis design, including analysis method (SAIGE‐GENE, followed by Fisher's method of combining p‐values) and case/control counts. (B) Quantile–quantile plot of rare variant aggregate testing meta‐analysis results. (C) Manhattan plot of rare variant aggregate testing meta‐analysis results. Any genes meeting the genome‐wide significance threshold (p < 2.5 × 10^−6^) are identified with their gene name. [file JCSM-17-e70293-s028.tif]

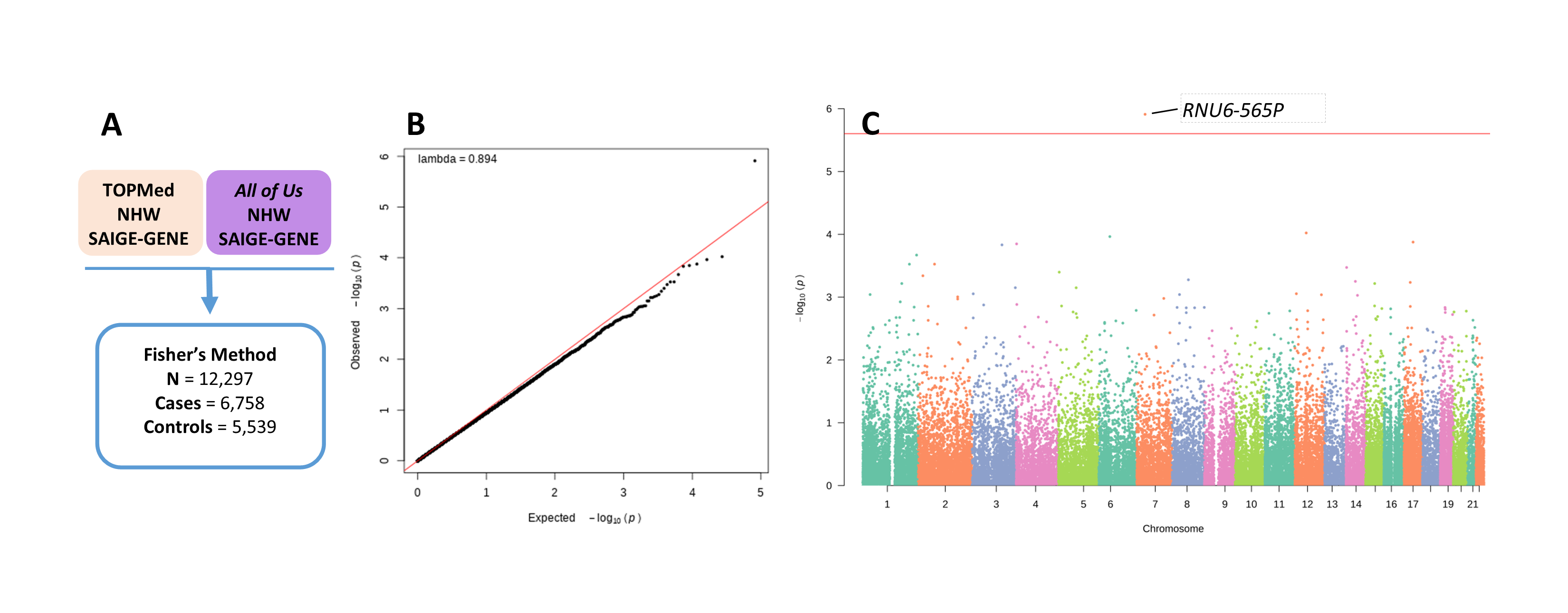

Supplement: Supplementary file 19 — Figure S17: Meta‐analysis of rare variant aggregate association testing results from non‐Hispanic white (NHW) participants from the Trans‐Omics for Precision Medicine (TOPMed) Initiative and All of Us Research Program. (A) Analysis design, including analysis method (SAIGE‐GENE, followed by Fisher's method of combining p‐values) and case/control counts. (B) Quantile–quantile plot of rare variant aggregate testing meta‐analysis results. (C) Manhattan plot of rare variant aggregate testing meta‐analysis results. Any genes meeting the genome‐wide significance threshold (p < 2.5 × 10^−6^) are identified with their gene name. [file JCSM-17-e70293-s024.tif]

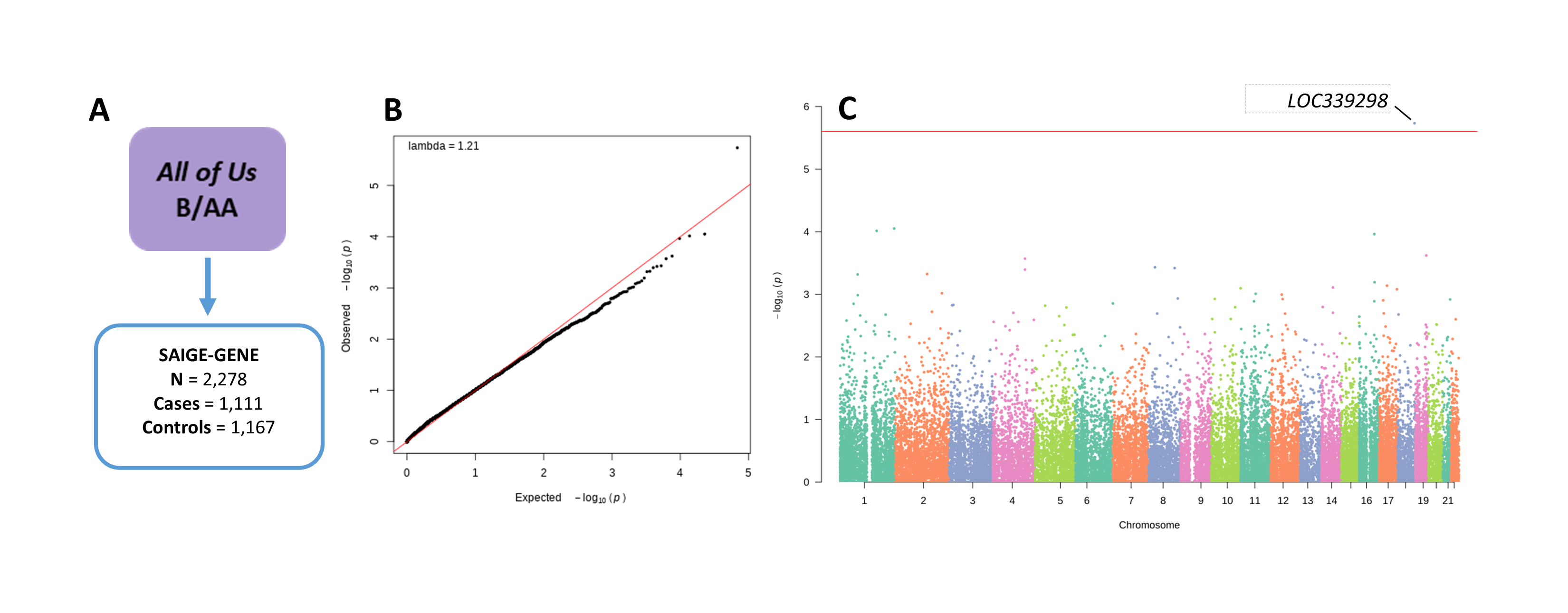

Supplement: Supplementary file 20 — Figure S18: Rare variant aggregate association testing results from Black/African–American (B/AA) participants from the All of Us research program. (A) Analysis design, including analysis method (SAIGE‐GENE) and case/control counts. (B) Quantile–quantile plot of rare variant aggregate testing meta‐analysis results. (C) Manhattan plot of rare variant aggregate testing meta‐analysis results. Any genes meeting the genome‐wide significance threshold (p < 2.5 × 10^−6^) are identified with their gene name. [file JCSM-17-e70293-s029.tif]

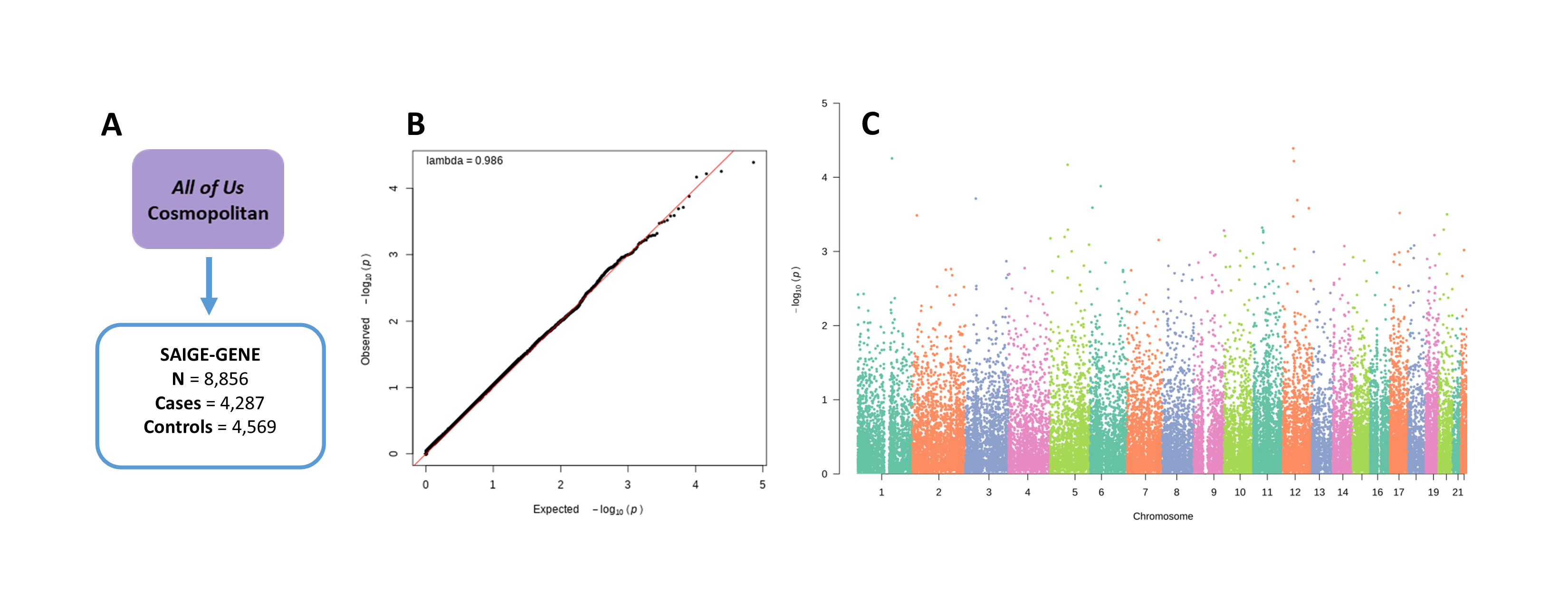

Supplement: Supplementary file 21 — Figure S19: Rare variant aggregate association testing results from all participants from the All of Us research program. (A) Analysis design, including analysis method (SAIGE‐GENE) and case/control counts. (B) Quantile–quantile plot of rare variant aggregate testing meta‐analysis results. (C) Manhattan plot of rare variant aggregate testing meta‐analysis results. Any genes meeting the genome‐wide significance threshold (p < 2.5 × 10^−6^) are identified with their gene name. [file JCSM-17-e70293-s025.tif]

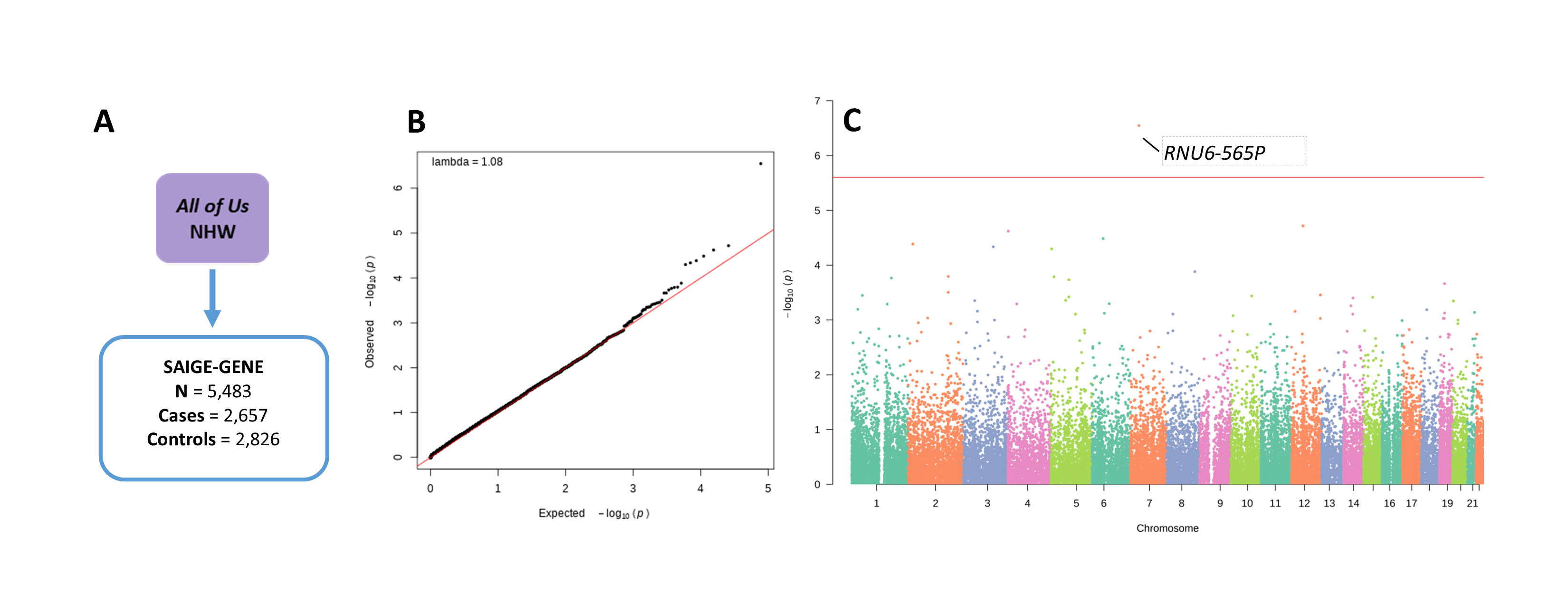

Supplement: Supplementary file 22 — Figure S20: Rare variant aggregate association testing results from non‐Hispanic white (NHW) participants from the All of Us research program. (A) Analysis design, including analysis method (SAIGE‐GENE) and case/control counts. (B) Quantile–quantile plot of rare variant aggregate testing meta‐analysis results. (C) Manhattan plot of rare variant aggregate testing meta‐analysis results. Any genes meeting the genome‐wide significance threshold (p < 2.5 × 10^−6^) are identified with their gene name. [file JCSM-17-e70293-s012.tif]

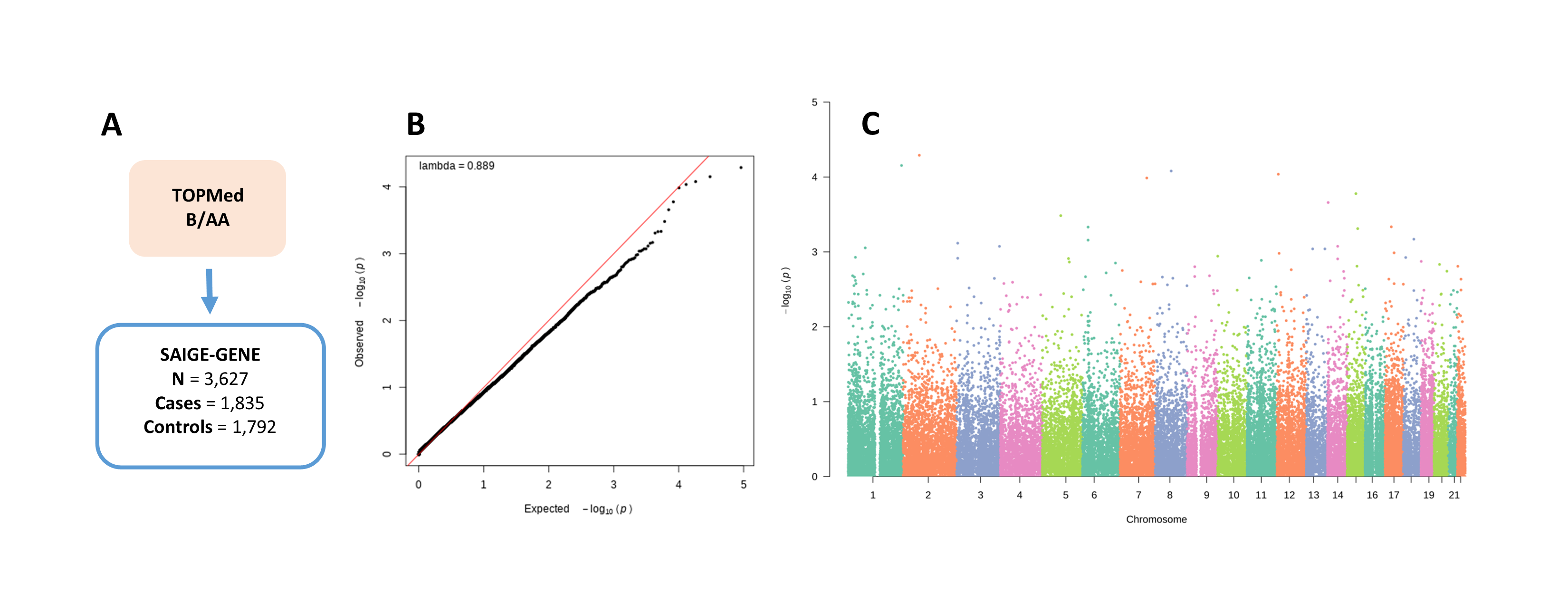

Supplement: Supplementary file 23 — Figure S21: Rare variant aggregate association testing results from Black/African–American (B/AA) participants from the Trans‐Omics for Precision Medicine (TOPMed) Initiative. (A) Analysis design, including analysis method (SAIGE‐GENE) and case/control counts. (B) Quantile–quantile plot of rare variant aggregate testing meta‐analysis results. (C) Manhattan plot of rare variant aggregate testing meta‐analysis results. Any genes meeting the genome‐wide significance threshold (p < 2.5 × 10^−6^) are identified with their gene name. [file JCSM-17-e70293-s013.tif]

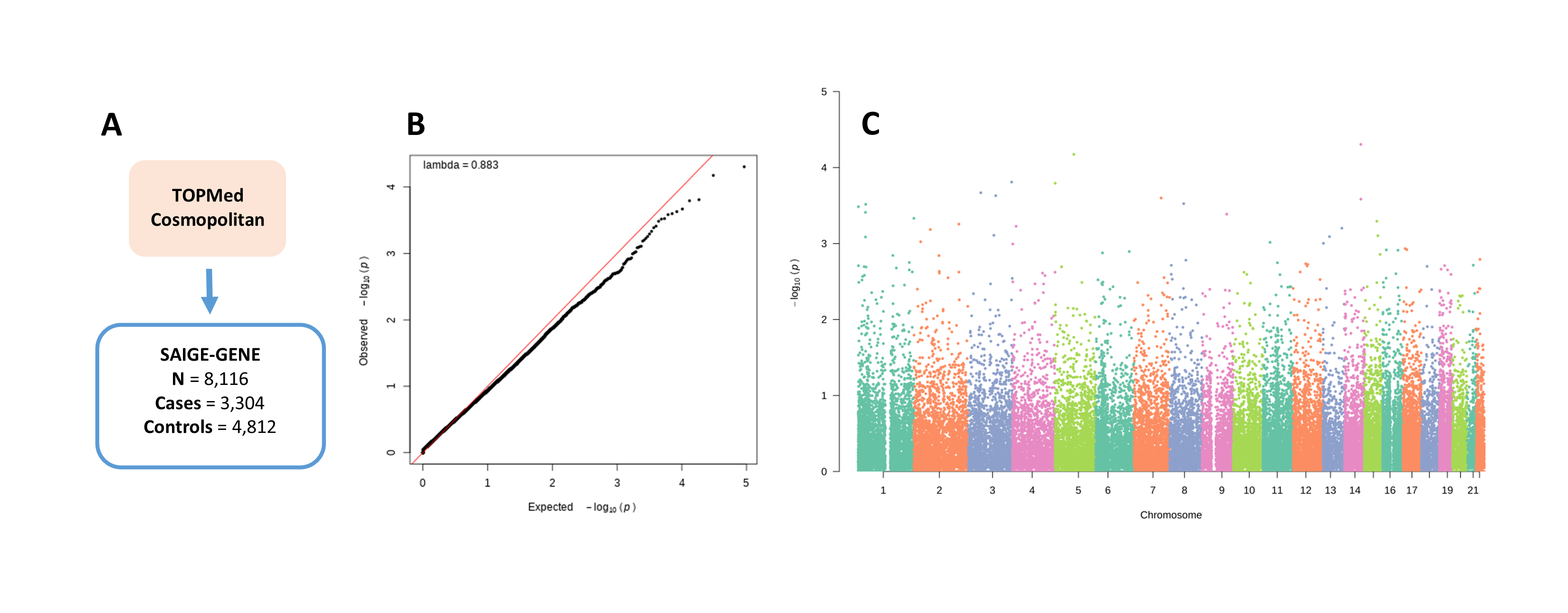

Supplement: Supplementary file 24 — Figure S22: Rare variant aggregate association testing results from all participants from the Trans‐Omics for Precision Medicine (TOPMed) Initiative. (A) Analysis design, including analysis method (SAIGE‐GENE) and case/control counts. (B) Quantile–quantile plot of rare variant aggregate testing meta‐analysis results. (C) Manhattan plot of rare variant aggregate testing meta‐analysis results. Any genes meeting the genome‐wide significance threshold (p < 2.5 × 10^−6^) are identified with their gene name. [file JCSM-17-e70293-s005.tif]

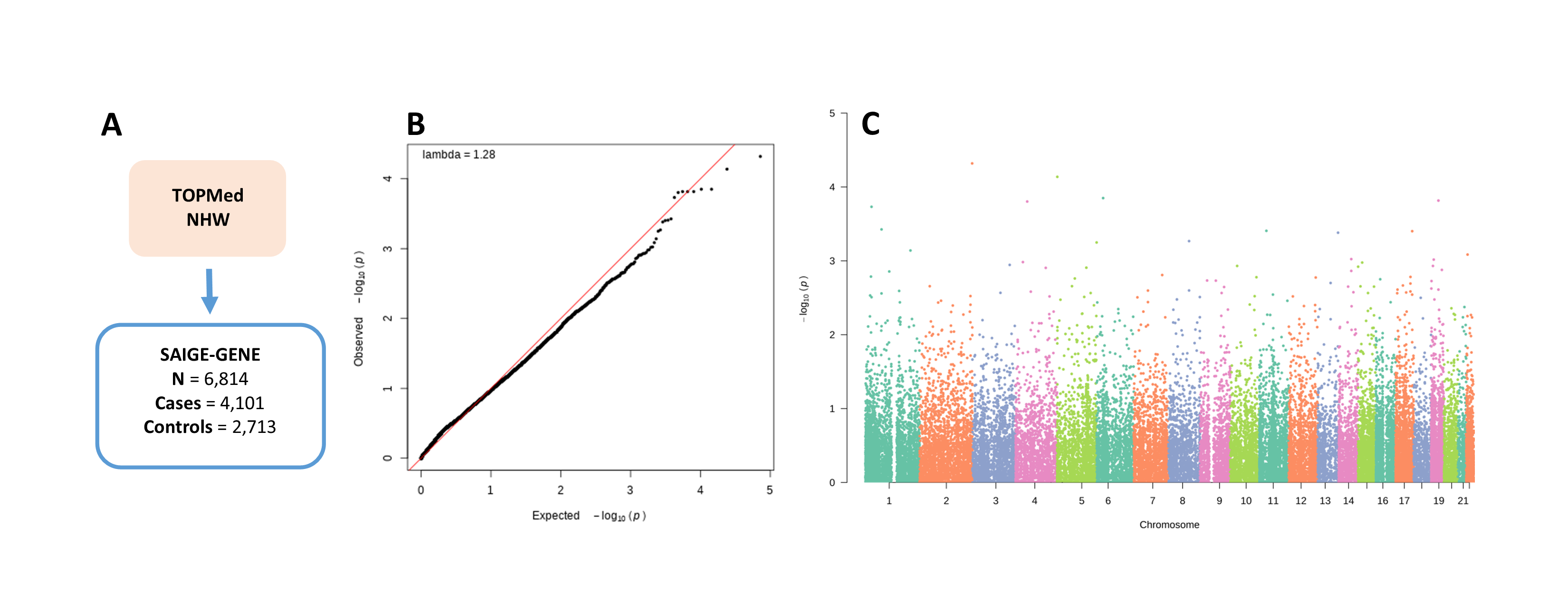

Supplement: Supplementary file 25 — Figure S23: Rare variant aggregate association testing results from non‐Hispanic White (NHW) participants from the Trans‐Omics for Precision Medicine (TOPMed) Initiative. (A) Analysis design, including analysis method (SAIGE‐GENE) and case/control counts. (B) Quantile–quantile plot of rare variant aggregate testing meta‐analysis results. (C) Manhattan plot of rare variant aggregate testing meta‐analysis results. Any genes meeting the genome‐wide significance threshold (p < 2.5 × 10^−6^) are identified with their gene name. [file JCSM-17-e70293-s008.tif]

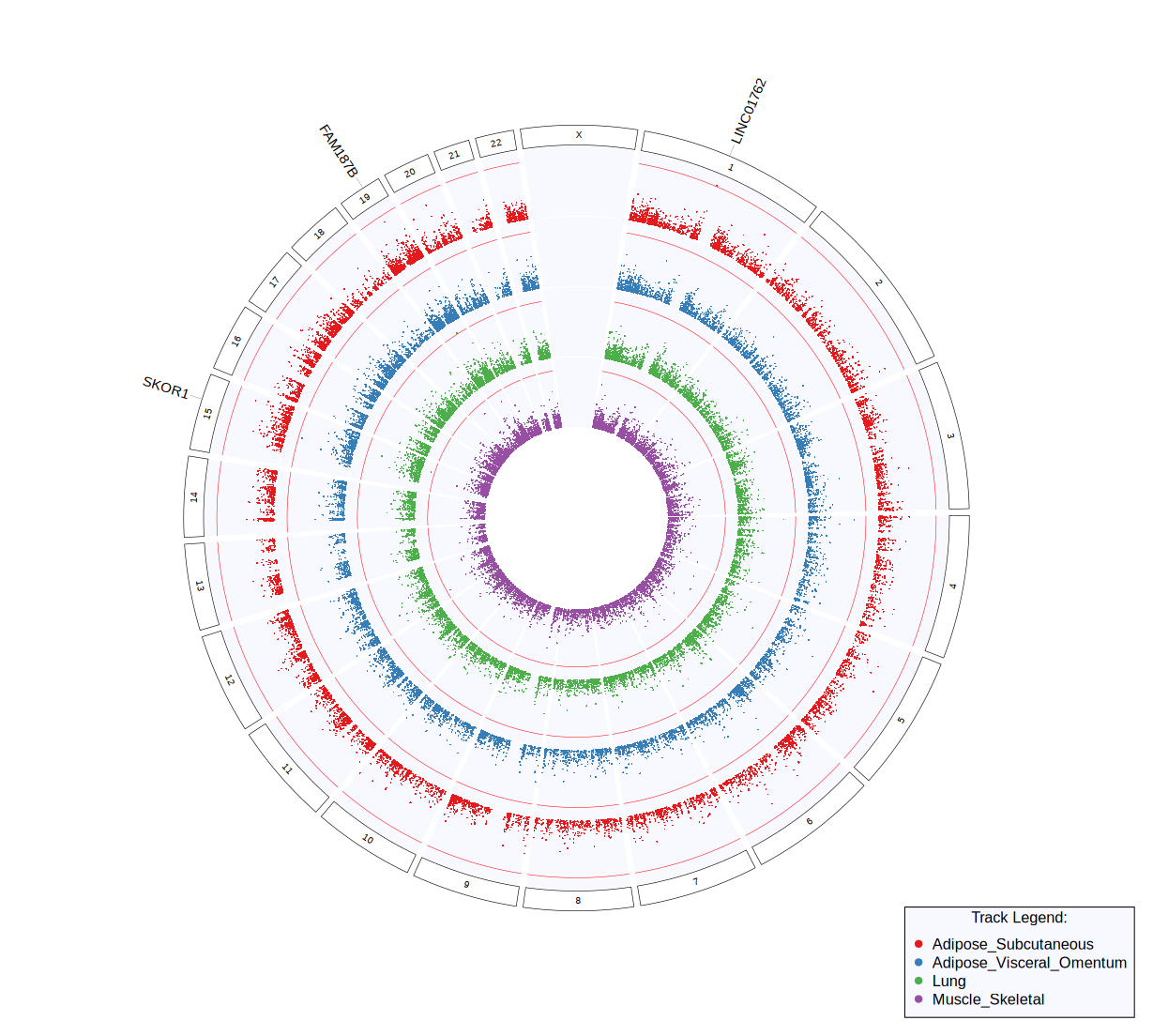

Supplement: Supplementary file 26 — Figure S24: Predicted genetically regulated gene expression effects of meta‐analysed single variant associations with weight loss in Black/African–American (B/AA) participants with COPD from MetaXcan. Genes meeting nominal genome‐wide significance (p < 10−5) are identified by name. [file JCSM-17-e70293-s020.png]

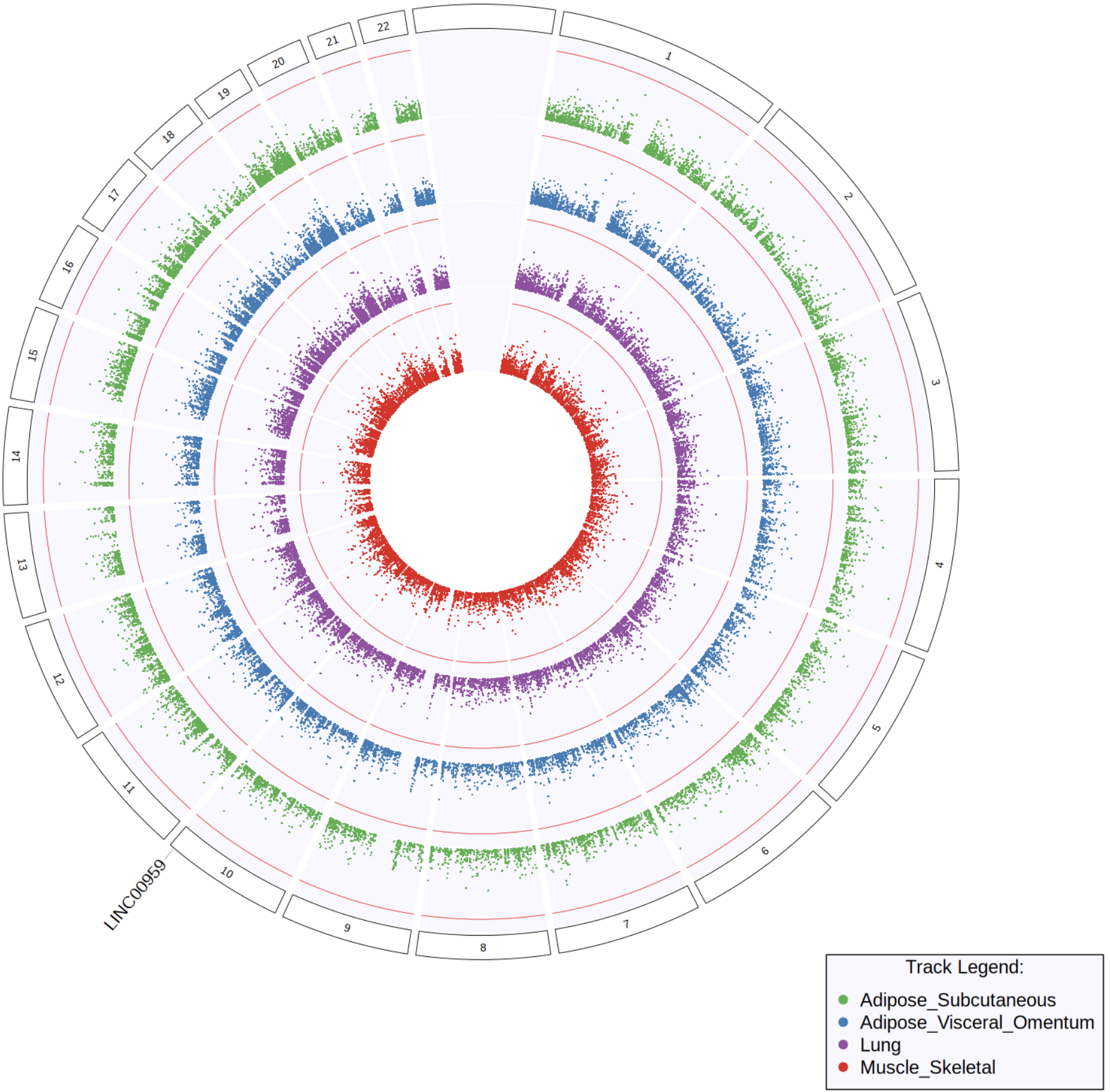

Supplement: Supplementary file 27 — Figure S25: Predicted genetically regulated gene expression effects of meta‐analysed single variant associations with weight loss in all (COSMO) participants with COPD from MetaXcan. Genes meeting nominal genome‐wide significance (p < 10−5) are identified by name. [file JCSM-17-e70293-s021.png]

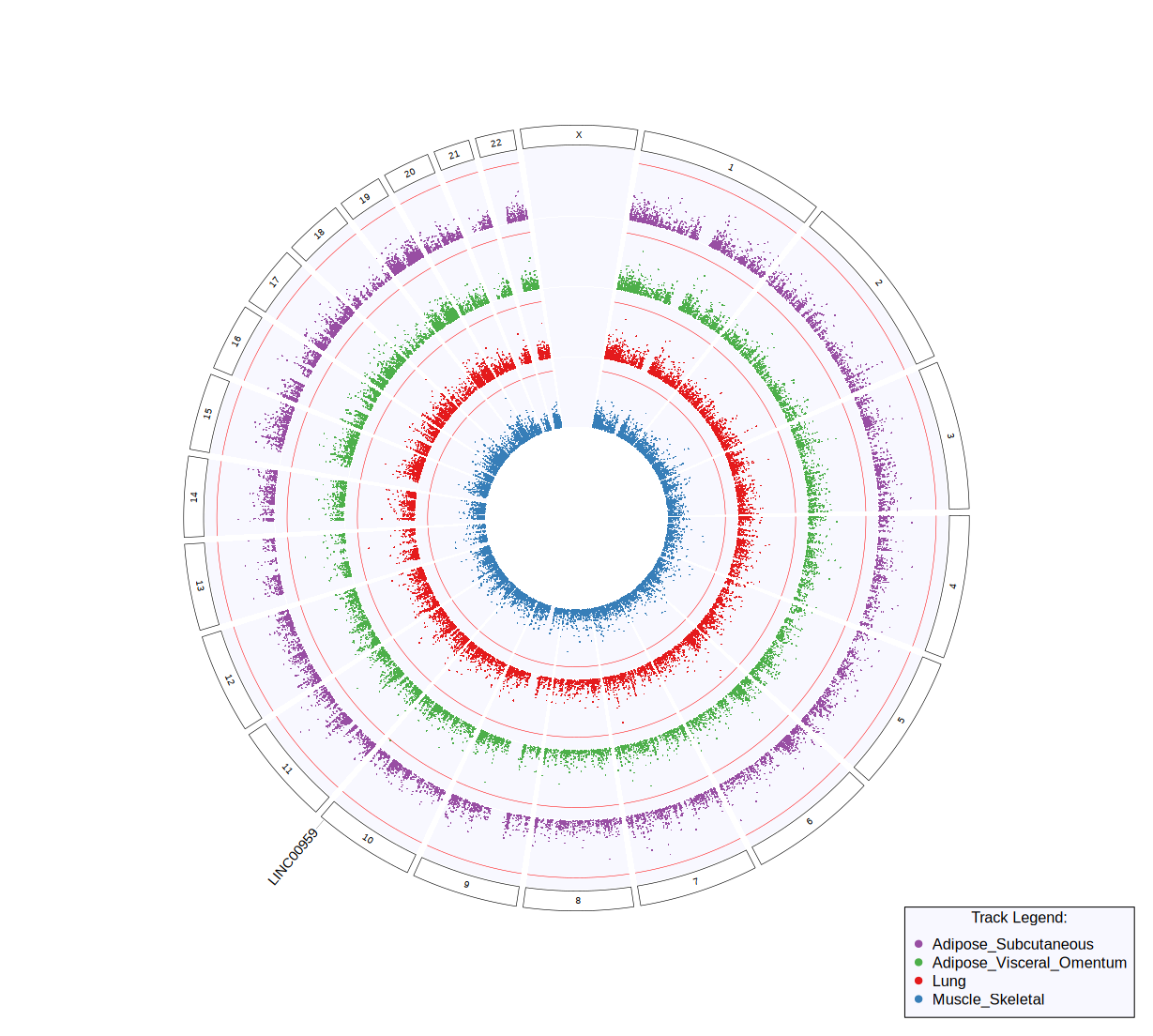

Supplement: Supplementary file 28 — Figure S26: Predicted genetically regulated gene expression effects of meta‐analysed single variant associations with weight loss in non‐Hispanic white (NHW) Participants with COPD from MetaXcan. Genes meeting nominal genome‐wide significance (p < 10−5) are identified by name. [file JCSM-17-e70293-s026.png]

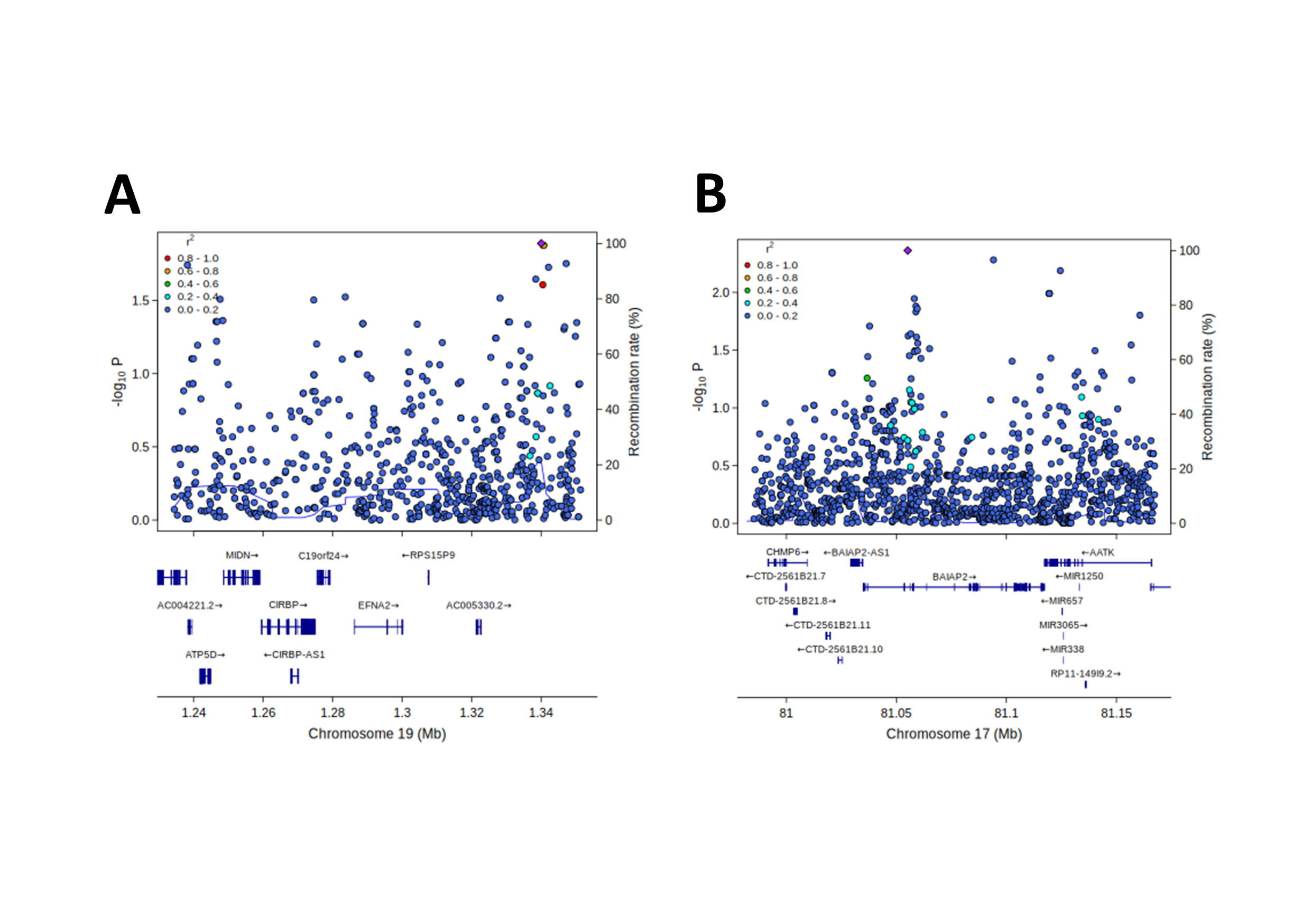

Supplement: Supplementary file 29 — Figure S27: Regional association plots of single variant associations with weight loss in COPD near previously implicated genes. Gene names are found along the bottom panel with exons represented by filled‐in boxes. P‐values are plotted on a negative log scale on the y‐axis, and each dot represents a variant. [file JCSM-17-e70293-s027.tif]
